# Identifying plant-derived antiviral alkaloids as dual inhibitors of SARS-CoV-2 main protease and spike glycoprotein through computational screening

**DOI:** 10.3389/fphar.2024.1369659

**Published:** 2024-07-17

**Authors:** Ramsha Yamin, Iqra Ahmad, Hira Khalid, Asia Perveen, Sumra Wajid Abbasi, Umar Nishan, Sheheryar Sheheryar, Arlindo Alencar Moura, Sarfraz Ahmed, Riaz Ullah, Essam A. Ali, Mohibullah Shah, Suvash Chandra Ojha

**Affiliations:** ^1^ Department of Biochemistry, Bahauddin Zakariya University, Multan, Pakistan; ^2^ Department of Biological Sciences, National University of Medical Sciences, Rawalpindi, Pakistan; ^3^ Department of Chemistry, Kohat University of Science & Technology, Kohat, Pakistan; ^4^ Department of Animal Science, Federal University of Ceara, Fortaleza, Brazil; ^5^ Wellman Centre for Photomedicine, Massachusetts General Hospital, Harvard Medical School, Boston, MA, United States; ^6^ Department of Pharmacognosy, College of Pharmacy, King Saud University, Riyadh, Saudi Arabia; ^7^ Department of Pharmaceutical Chemistry, College of Pharmacy, King Saud University, Riyadh, Saudi Arabia; ^8^ Department of Infectious Diseases, The Affiliated Hospital of Southwest Medical University, Luzhou, China

**Keywords:** alkaloids, main protease, spike glycoprotein, secondary metabolites, SARS-CoV-2

## Abstract

COVID-19 is currently considered the ninth-deadliest pandemic, spreading through direct or indirect contact with infected individuals. It has imposed a consistent strain on both the financial and healthcare resources of many countries. To address this challenge, there is a pressing need for the development of new potential therapeutic agents for the treatment of this disease. To identify potential antiviral agents as novel dual inhibitors of SARS-CoV-2, we retrieved 404 alkaloids from 12 selected medicinal antiviral plants and virtually screened them against the renowned catalytic sites and favorable interacting residues of two essential proteins of SARS-CoV-2, namely, the main protease and spike glycoprotein. Based on docking scores, 12 metabolites with dual inhibitory potential were subjected to drug-likeness, bioactivity scores, and drug-like ability analyses. These analyses included the ligand–receptor stability and interactions at the potential active sites of target proteins, which were analyzed and confirmed through molecular dynamic simulations of the three lead metabolites. We also conducted a detailed binding free energy analysis of pivotal SARS-CoV-2 protein inhibitors using molecular mechanics techniques to reveal their interaction dynamics and stability. Overall, our results demonstrated that 12 alkaloids, namely, adouetine Y, evodiamide C, ergosine, hayatinine, (+)-homoaromoline, isatithioetherin C, N,alpha-L-rhamnopyranosyl vincosamide, pelosine, reserpine, toddalidimerine, toddayanis, and zanthocadinanine, are shortlisted as metabolites based on their interactions with target proteins. All 12 lead metabolites exhibited a higher unbound fraction and therefore greater distribution compared with the standards. Particularly, adouetine Y demonstrated high docking scores but exhibited a nonspontaneous binding profile. In contrast, ergosine and evodiamide C showed favorable binding interactions and superior stability in molecular dynamics simulations. Ergosine demonstrated exceptional performance in several key pharmaceutical metrics. Pharmacokinetic evaluations revealed that ergosine exhibited pronounced bioactivity, good absorption, and optimal bioavailability. Additionally, it was predicted not to cause skin sensitivity and was found to be non-hepatotoxic. Importantly, ergosine and evodiamide C emerged as superior drug candidates for dual inhibition of SARS-CoV-2 due to their strong binding affinity and drug-like ability, comparable to known inhibitors like N3 and molnupiravir. This study is limited by its *in silico* nature and demands the need for future *in vitro* and *in vivo* studies to confirm these findings.

## 1 Introduction

On 31 December 2019, in China, a cluster of pneumonia cases was identified in people associated with the seafood wholesale market in Wuhan, Hubei Province. On 7 January 2020, it was confirmed by Chinese health authorities that this cluster is related to a novel coronavirus, COVID-19 (She et al., 2020). COVID-19 was considered the ninth-deadliest pandemic that spread through either direct or indirect contact with infected individuals. Cough, flu, fever, chest pain, shortness of breath, cold, tiredness, and gastrointestinal problems are some common symptoms reported in COVID-19 patients (Nobel et al., 2020; Murugesan et al., 2021). The binding of the coronavirus to the lung epithelial cells caused the respiratory failure of the infected person and is the major cause of death (Zhang Y. et al., 2020).

Regarding the possible treatments for this disease, research has mainly focused on the invention of potential vaccines or designing potential drugs (Liu et al., 2020; Jaan et al., 2021; Shah et al., 2024). So far, not even a single drug has been discovered or formulated specifically for the treatment of COVID-19 (Vegad et al., 2023). Some FDA-approved antiviral drugs, like remdesivir, chloroquine, ribavirin, hydroxychloroquine, favipiravir, and lopinavir, among others, were found to provide partial relief from the deadly virus (Jean and Hsueh, 2020; Wu et al., 2020). However, none of these drugs can mitigate its pathogenesis, and their associated toxicity also brings several complications (Murugesan et al., 2021).

Besides these available drugs, efforts are directed toward discovering and designing new drugs from natural sources with the least toxicity and the most efficacy by inhibiting the potential targets of an organism. Computer-aided drug design techniques are now routinely used in drug discovery to screen a multitude of compounds to identify and develop potential leads against infectious bacteria or viruses, thus reducing the labor and cost of the process. Most antiviral drugs that target only a particular viral enzyme have reduced effectiveness due to rapid mutational changes in the viral target. To overcome this problem, the discovery of multitargeted drugs provided a suitable alternative. The COVID-19 virus has extensive mutational changes, and multitargeted drug design is the best approach to overcome these mutational changes and control the pathogenesis of COVID-19 (Naik et al., 2020).

The main protease is the central enzyme involved in coronavirus replication and transcription (Jin et al., 2020a) and is hence considered a potent drug target in the drug discovery against COVID-19 (Sisakht et al., 2021). Spike glycoproteins, found on the outer surface of coronavirus, recognize the host cell surface receptor angiotensin-converting enzyme 2 (ACE-2) (Zhang H. et al., 2020). ACE-2 is the lead cellular receptor for coronavirus entry that initiates the infectious process (Li et al., 2003). The recognition between viral spike glycoprotein and host receptor-binding domains is necessary for the fusion of the membranes of the viral and host cells. The importance of this protein for the virus makes it an important drug target, and it has been exploited in several studies for this purpose (Maurya et al., 2022; Adedotun et al., 2023; Debnath et al., 2023).

Antiviral plants are an important source for potential drug candidates against COVID-19. Plant-based medicines are less toxic and more effective for the treatment of a number of ailments than synthetic medicines. Several plants are famous for their antiviral potential (Mukhtar et al., 2008). One of the most popular and safe strategies for COVID-19 drug design is through plant-derived secondary metabolites. Different studies suggest the potential of plant-derived natural products and phytochemical extracts as anti-COVID drugs (Boukhatem and Plants, 2020; Khaerunnisa et al., 2020; Khan et al., 2020; Shree et al., 2022).

Alkaloids are one of the most important groups of plant-derived secondary metabolites and possess several biological activities, such as antiviral, antimicrobial, antibacterial, and antifungal activities (Zhang et al., 2018; Xu et al., 2019; Abookleesh et al., 2022). Recent studies have shown the anti-COVID-19 potential of alkaloids (Majnooni et al., 2021; Ghosh et al., 2022; Bileşikler et al., 2020), marking them as attractive natural therapeutic agents. In this study, we have selected the important antiviral plant-derived alkaloids to be screened against important COVID-19 drug targets, namely, the main protease and spike glycoprotein. Our findings showed that plant-derived alkaloids are worthy of studies oriented toward drug design against COVID-19.

## 2 Materials and methods

### 2.1 Retrieval of antiviral ligands

In this study, we focused on the identification and analysis of plant-derived antiviral alkaloids with therapeutic potential against SARS-CoV-2. In this regard, 12 medicinal plants with known antiviral potential were selected, and the reported alkaloids from these plants were collected for analysis. A total of 40 alkaloids were obtained from *Cissampelos pareira* L. [Menispermaceae], 64 from *Toddalia asiatica* (L.) Lam. [Rutaceae], and 58 from *Alstonia scholaris* (L.) R.Br. [Apocynaceae], 9 from *Rhizophora apiculata* Blume [Rhizophoraceae], 28 from *Peganum harmala* L. [Nitrariaceae], 4 from *Rhizophora mucronata* Poir. [Rhizophoraceae], 38 from *Isatis indigotica* Fort. [Brassicaceae], 33 from *Waltheria indica* L. [Malvaceae], 36 from *Argemone mexicana* L. [Papaveraceae], 25 from *Evodia rutaecarpa* (Juss.) Benth. [Rutaceae], 58 from *Dictamnus dasycarpus* Turcz. [Rutaceae], and 10 from *Moringa oleifera* Lam. [Moringaceae]. As a result, an *in-house* library of 403 alkaloids was generated and used for further analysis (Supplementary Table S1).

### 2.2 Ligand preparation and optimization

The structures of the collected alkaloids were either retrieved from PubChem, constructed using CS ChemDraw, or saved in mol format. The library was prepared in Molecular Operating Environment (MOE), and energy minimization was performed using the default parameters. The N3 inhibitor and molnupiravir were used as standards against the main protease and spike glycoprotein, respectively.

### 2.3 Receptor preparation and active site allocation and docking analysis

The crystal structures of the coronavirus spike receptor-binding domain in complex with its receptor ACE2 (PDB ID: 6LZG) and main protease (PDB ID: 6LU7) were downloaded from the Protein Data Bank (https://www.rcsb.org/). All the attached water molecules, inhibitors, and repeated chains were removed to avoid any complications during the ligand–protein docking process. The attached inhibitors, water molecules, and repeated chains may create obstructions in ligand and protein interactions. Chain A of the main protease and chains A and B of the S-protein were used for docking purposes. Energy minimization and protonation of target proteins were carried out in MOE using default parameters (Shah et al., 2021; Shah et al., 2023). For both the initial docking of alkaloids and the subsequent docking of reference compounds, the same protocol was followed for the selection of the active site residues. The active site was selected based on its large size and the presence of amino acid residues that are involved in the ligand–protein interaction. Dummy atoms were created by selecting the dummy option, and atoms and backbones were isolated in the site finder tool of MOE. Notably, this largest active site remained consistent across repeated docking sessions, confirming the reproducibility of the site selection. Molecular docking was carried out in MOE using the induced-fit docking protocol. Dummy atoms were selected in the site module so that these atoms of the target protein may be involved in the molecular docking process with the ligand library. Five conformational poses were selected to be retained in the result file.

### 2.4 Docking validation

Using re-docking and superimposition techniques, we implemented a strict validation approach to guarantee accuracy and the screening’s reliance on docking (Shah et al., 2023; Ahmad et al., 2024). Complexes of the main protease with its co-crystallized ligand, as well as the complexes of docked and re-docked standards of the S-protein, were prepared in MOE software, and later on, their superimposition was done in PyMOL software, and their root mean square deviation (RMSD) values were noted in order to validate our docking protocol.

### 2.5 MD simulation

Molecular dynamics (MD) simulations of 6LU7 and 6LZG protein complexes were performed with our best ligands. The simulations were performed for 100 ns in a solvent environment of TIP3P water within a 12 Å water box, utilizing the Amber 20 tool (Ponder and Case, 2003; Wang et al., 2004). Pre- and post-simulation processing were performed using the previously described method (Abbasi et al., 2016). Briefly, for ligands, GAFF force was used, whereas ff99SB was used for selected proteins. The LEaP module of the Amber suit was used to record the topologies of ligands and proteins. To neutralize the systems, three sodium ions were added to 6LU7 and 26 sodium ions to 6LZG-bound ligands. A TIP3P water molecule box was used to solvate all the studied systems, with an 8.0 Å spacing around the docked complexes and proteins. Periodic boundary conditions were implemented to mitigate edge effects. The structural integrity of the docked complexes was verified using Amber. Minimization of the solvated systems was achieved through 1,000 steps of the conjugate gradient method, followed by 1,500 steps of the steepest descent method, with an 8 Å cutoff distance. These simulations were carried out in the NVT ensemble, using the SHAKE algorithm to maintain bond constraints. Initial system preparation involved gradual heating and volume adjustment for more than 2 ps, reaching equilibrium at 300 K and 1 atm. The Berendsen coupling method was used for temperature regulation. Initial equilibration at 300 K for 100 ps was conducted before the production run. The equilibration phase involved an interchange of kinetic and potential energy. During equilibration, total energy remained nearly constant, whereas potential and kinetic energies varied. Atom positions were recorded every 100 ps during the simulation to generate trajectory files. Post-simulation, these trajectories were examined for parameters such as RMSD, root mean square fluctuation (RMSF), radius of gyration (RoG), and solvent accessible surface area (SASA) analysis using the PTRAJ module of Amber. The trajectories of the simulations were recorded and stored for periodic inspection. This comprehensive approach allowed for the exploration of ligand–protein interactions and their stability in a simulated physiologic context.

### 2.6 Computation of binding free energy using MM-GBSA and MM-PBSA

The binding free energy for the selected ligands and reference drugs docked with 6LU7 and 6LZG was estimated using two methods: molecular mechanics–generalized Born surface area and molecular mechanics Poisson–Boltzmann surface area (Kollman et al., 2000). As reported earlier, to compute the binding free energy, 1,000 snapshots were taken during the MD simulation trajectory using the same techniques. The net energy of the system was estimated using the following equation:

ΔGBinding=ΔGComplex– ΔGReceptor– ΔGLigand.

The process entails computing various energy types, including van der Waals, electrostatic, and internal energies derived from molecular mechanics. Additionally, it considers the influence of the nonpolar component as well as the polar aspect of solvation energy.

### 2.7 Bioactivity score analysis

The bioactivity score analysis of the top 12 ligands was performed through the Molinspiration Cheminformatics tool (https://www.molinspiration.com/). It comprises different parameters like G protein-coupled receptor ligand, nuclear receptor ligand, ion channel modulator, enzyme inhibition, kinase inhibition, and enzyme inhibition (Murugesan et al., 2021).

### 2.8 Drug-likeness analysis

For a compound to be selected as a potential drug candidate, it is important to follow its so-called drug-like properties. The drug-likeness and physiochemical properties of the top 12 ligands were studied through the Swiss ADME online server (http://www.swissadme.ch/). Drug-likeness based on different rules, like Lipinski (Lipinski et al., 1997), Veber (Veber et al., 2002), Ghose (Ghose et al., 1999), and Egan (Egan et al., 2000), depends on physicochemical properties.

### 2.9 ADMET analysis

ADMET (absorption, distribution, metabolism, excretion, and toxicity) properties of top lead alkaloids were evaluated through the pkCSM online tool (Pires et al., 2015) for pharmacokinetics analysis (https://biosig.lab.uq.edu.au/pkcsm/). Canonical smiles of ligands were used as an input in pkCSM, and the obtained results were analyzed accordingly.

## 3 Results and discussion

### 3.1 Active site prediction

The active site of the main protease is present around the amino acids His41 and Cys145 in the S1 subpocket as the catalytic dyad. These residues are significant for the effective binding of drug candidates and for inhibiting the hydrolytic activity of the main protease. Binding with Glu166 is pivotal in keeping it in active conformation (Al-Rabia et al., 2021; Omar et al., 2023). Furthermore, the binding with Thr45, Met49, Phe140, Met165, Glu166, Asp187, Asn142, His172, Arg188, and Gln189 is responsible for the grid opening of the catalytic site (Banerjee et al., 2021). We selected the active site of the main protease by considering these catalytically significant residues. The selected active sites of the main protease included Thr25, Thr26, Leu27, His41, Val42, Cys44, Thr45, Ser46, Met49, Pro52, Tyr59, Phe140, Leu141, Asn142, Gly143, Ser144, Cys145, His163, His164, Met165, Glu166, Leu167, Pro168, His172, Asp187, Arg188, Gln189, and Thr190.

Among the predicted active sites of spike glycoprotein by MOE Site Finder, the active sites selected in this study include Leu5, Gln96, Gln98, Ala99, Gln102, Tyr196, Tyr202, Trp203, Gly205, Asp206, Tyr207, Glu208, Val209, Arg219, Phe390, Leu391, Leu392, Arg393, Asn394, Ala396, Asn397, Glu398, Ser511, Arg514, Lys562, Pro565, Trp566, and Lys582. Among these residues, some were like the potential catalytic sites and favorable interacting residues of spike glycoprotein (Khan et al., 2022; Medina-Barandica et al., 2023), whereas other residues were part of the SARS-CoV-2 spike protein-ACE-2 interface. Targeting the interface residues limits the binding of ACE-2 with spike glycoprotein (Ortega et al., 2020; Thangavel et al., 2021).

### 3.2 Ligand binding affinity with receptors

To find potential drug candidates for the treatment of deadly SARS-CoV-2, molecular docking of 404 natural alkaloids was performed against the main protease and S-protein, taking N3 and molnupiravir as standards, respectively. The docking analysis of the ligands with the receptor proteins demonstrated that most of the lead metabolites belong to *W*. *indica* L. [Malvaceae], *R. apiculate* Blume [Rhizophoraceae], *E*. *rutaecarpa* (Juss.) Benth. [Rutaceae], *C*. *pareira* L. [Menispermaceae], *I. indigotica* Fort. [Brassicaceae], *M*. *oleifera* Lam. [Moringaceae], and *T*. *asiatica* (L.) Lam. [Rutaceae] and show a significant binding affinity with the target proteins of COVID-19. Of the alkaloids that showed dual inhibitory potential, the top 12 (namely, adouetine Y, evodiamide C, ergosine, hayatinine, (+)-homoaromoline, isatithioetherin C, N,alpha-L-rhamnopyranosyl vincosamide, pelosine, reserpine, toddalidimerine, toddayanis, and zanthocadinanine) with a higher docking score than both standards were selected for further analysis (Supplementary Tables S2 and S3). N3, a Michael inhibitor designed in 2005 by Rao and coworkers, has been extensively validated for its efficiency in inhibiting the proteolytic activity of SARS-CoV-1 and MERS-CoV main proteases (Yang et al., 2005). Later, it was shown to bind and irreversibly inhibit SARS-CoV-2 M^pro^ as described by Yang and colleagues (Jin et al., 2020b). Therefore, N3 was selected as the standard for the main protease. The reason for selecting molnupiravir as a standard was that the literature survey showed the benefits of using it for the treatment of COVID-19 patients. Molnupiravir has been shown to reduce the risk of serious infections, leading to a 50% reduction in the chances of hospitalization and the death of patients (Thorlund et al., 2022). Paxlovid, the first oral anti-COVID drug recently licensed by the FDA, was also used as the standard for the main protease. Paxlovid has two components, namely, nirmatrelvir and ritonavir. Nirmatrelvir is a primary inhibitor of the main protease produced by Pfizer, whereas ritonavir increases its exposure to a level effective against SARS-CoV-2 (Tay et al., 2020).

**FIGURE 1:**
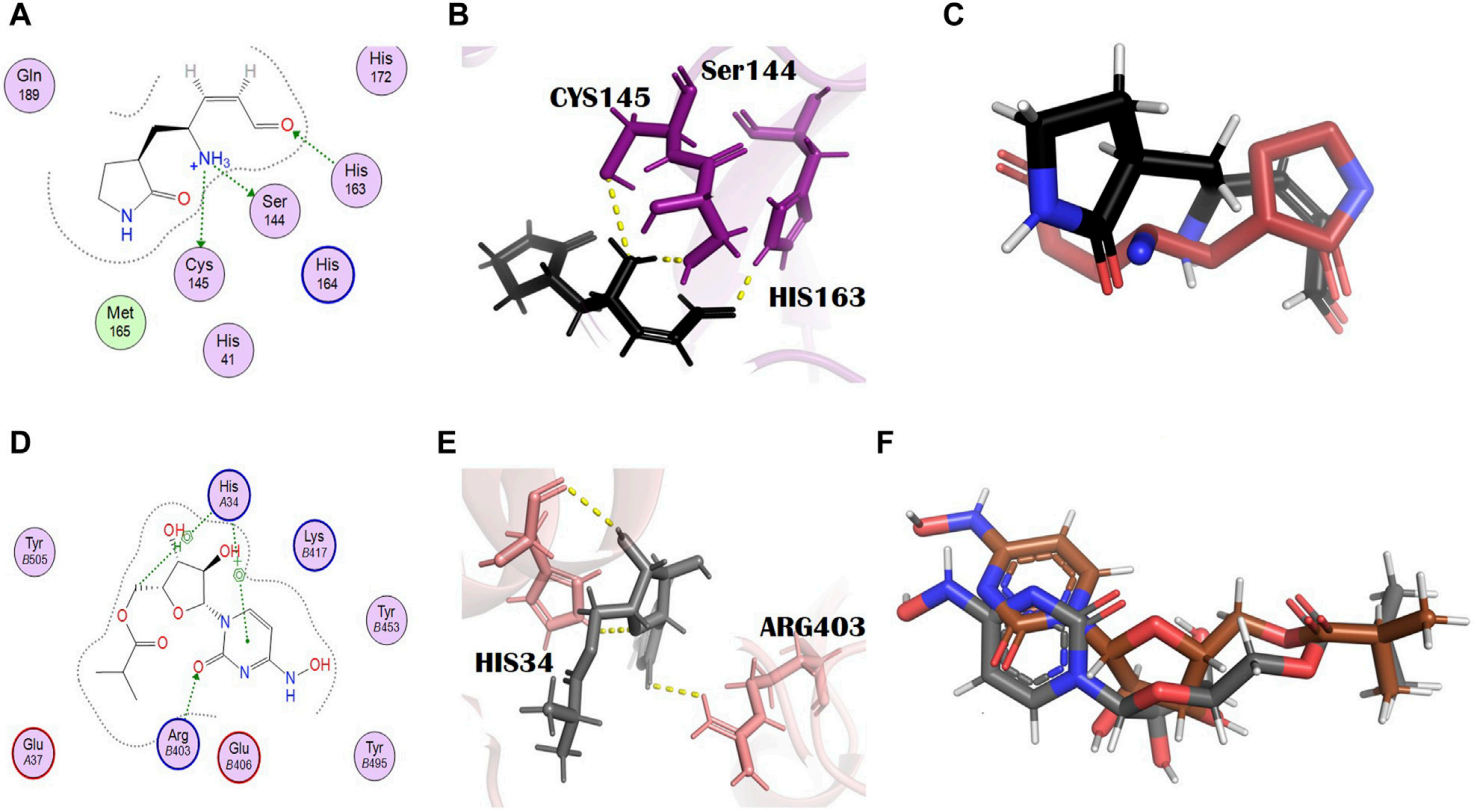
Molecular interaction analysis and superimposition of N3 and molnupiravir. **(A)** 2D interaction representation of N3 and **(D)** molnupiravir, **(B)** 3D interaction representation of N3 (black) with the main protease (purple) and **(E)** molnupiravir (gray) with 6LZG (salmon), **(C)** superimposition of co-crystalized N3 (red) from PDB 6LU7 with docked N3 (black), and **(F)** superimposition of docked molnupiravir (gray) with re-docked molnupiravir (brown).

N3, nirmatrelvir, and molnupiravir were docked against their respective proteins, and their docking scores were −4.76, −6.31, and −5.44, respectively. The docking scores of all selected dual inhibitors were higher than these standards. N3 formed three hydrogen bonds with SER144, CYS145, and HIS163 with bond distances of 2.0, 3.3, and 2.0 Å, respectively (Figures 1A, B). Nirmatrelvir interacted with the main protease with various bonds, including four H-donor interactions with Asn142, Gly143, and Gly189 (two H bonds), having bond distances of 2.88, 3.05, 2.69, and 2.60 Å, respectively. It formed three alkyl interactions with Met49, Cys145, and Pro168 with bond distances of 4.87, 5.23, and 4.52, respectively (Supplementary Figure S1). Molnupiravir formed one H bond with Arg403 and two H bonds with HIS34, with bond lengths of 2.96 Å, 3.73 Å, and 3.72 Å and bond energies of −1.2 kcal/mol, −0.6 kcal/mol, and −1.5 kcal/mol, respectively (Figures 1D, E).

### 3.3 Binding affinity and protein–ligand interactions

In general, the negative docking score of a compound is directly proportional to its binding affinity with the target protein. Studies of the interactions between proteins and their ligands are crucial for the interpretation of biological systems (Mishra et al., 2021). The protein–ligand complex stabilized due to these interactions (Muhammad et al., 2020; Shah et al., 2021). In this study, after performing the molecular docking of the ligands with the receptor proteins, the 2D interactions were inspected through MOE.

#### 3.3.1 Adouetine Y

**FIGURE 2:**
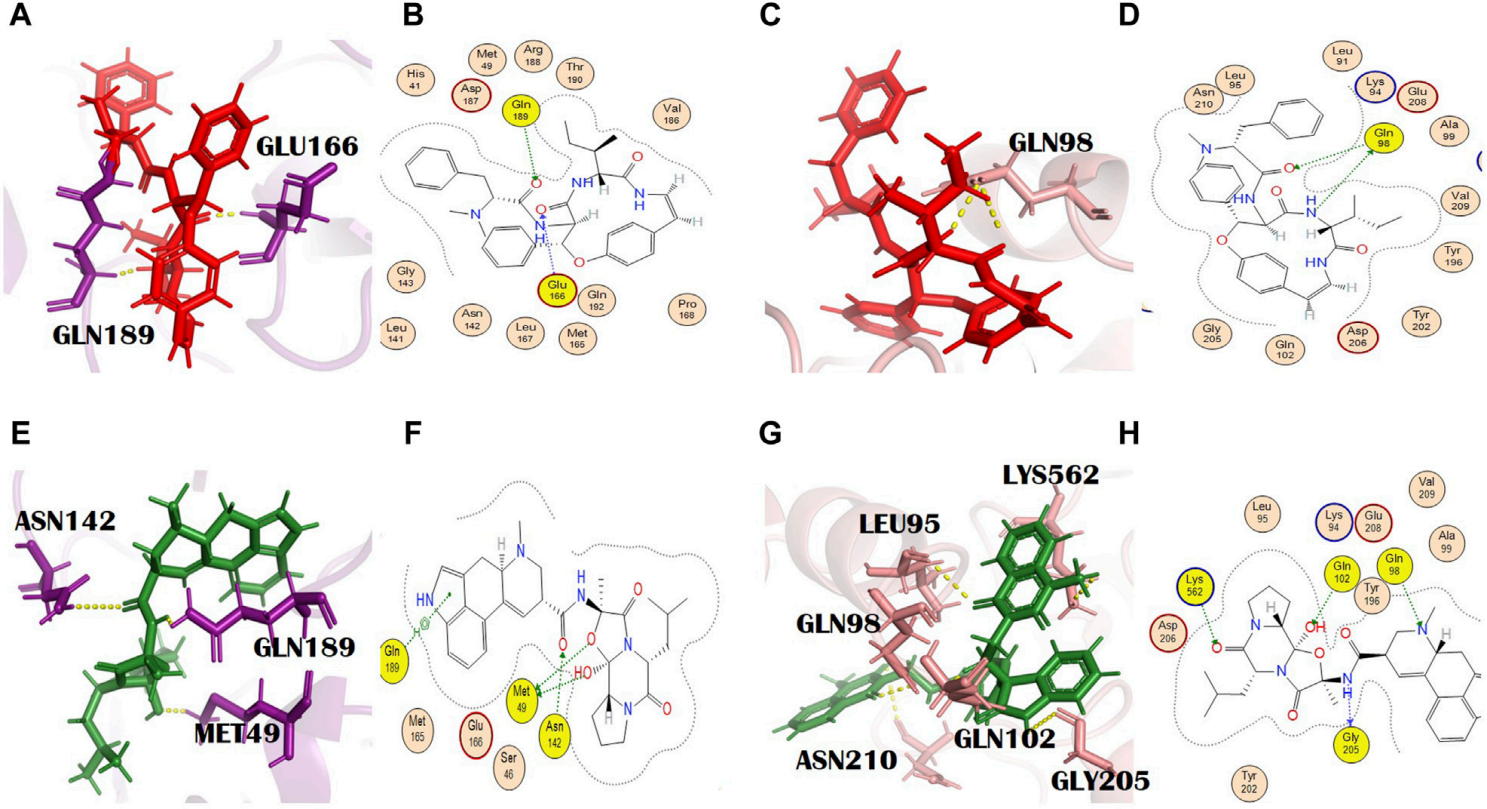
**(A)** 3D and **(B)** 2D molecular interactions of adouetine Y (red) with the main protease residues (purple), **(C)** 3D and **(D)** 2D molecular interactions of adouetine Y (red) with spike glycoprotein residues (salmon), **(E)** 3D and **(F)** 2D molecular interactions of ergosine (green) with the main protease (purple), **(G)** 3D and **(H)** 2D molecular interactions of ergosine (green) with spike glycoprotein (salmon).

Adouetine Y belongs to the cyclopeptide class of alkaloids (Giacomelli et al., 2012). (IUPAC name: (2S)-N-[(3R,4S,7S,10Z)-7-[(2S)-butan-2-yl]-5,8-dioxo-3-phenyl-2-oxa-6,9-diazabicyclo [10.2.2] hexadeca-1(14),10,12,15-tetraen-4-yl]-2-(dimethylamino)-3-phenylpropanamide). Its molecular formula is C34H40N4O4. The docking scores of adouetine Y with the main protease and spike glycoprotein were −8.40 kcal/mol and −8.66 kcal/jol, respectively (Supplementary Tables S2 and S3). It formed two H bonds with the COVID-19 main protease. The residues of the main protease that was involved in the formation of H bonds were Glu166 and Gln189 (Figures 2A, B). Glu166 formed an H bond of the H-acceptor type at a distance of 2.86 with an energy of −2.8. Gln189 is involved in the formation of the H bond, H-acceptor type, at a distance of 2.39 with −1.7 energy. This binding will favor the grid opening of the active site while keeping the main protease in its active conformation (Omar et al., 2023). It formed two H bonds with spike glycoprotein. The spike glycoprotein residue that formed the H bond with adouetine Y was Gln98. Gln98 formed two H bonds (H-donor and H-acceptor) at distances of 3.10 and 2.66 with energies of −2.66 and −1.2, respectively (Figures 2C, D). Adouetine Y showed the strongest binding affinity and was isolated from *W*. *indica* L. [Malvaceae]. This plant exhibited various pharmacologic activities like anti-cataract, anti-cancer, sedative, haematinic, anti-inflammatory, antifungal, analgesic, anti-diabetic, aphrodisiac, antibacterial, and antiviral activities (Nirmala and Sridevi, 2021). Furthermore, *W*. *indica* L. [Malvaceae] was found to inhibit rotavirus and human immunodeficiency virus (HIV) protease, thus having the potential to be used as an antiviral plant against SARS-CoV-2 (Zongo et al., 2013).

#### 3.3.2 Ergosine

Ergosine is an ergot alkaloid. Molecular formula: C30H37N5O5 and IUPAC name: (6*aR*,9*R*)-*N*-[(1*S*,2*S*,4*R*,7*S*)-2-hydroxy-4-methyl-7-(2-methylpropyl)-5,8-dioxo-3-oxa-6,9-diazatricyclo [7.3.0.0^2,6^] dodecan-4-yl]-7-methyl-6,6*a*,8,9-tetrahydro-4*H*-indolo[4,3-fg] quinoline-9-carboxamide. The total alkaloidal extract of *R*. *apiculata* Bl. [Rhizophoraceae], including ergosine, showed inhibitory potential against alpha-glucosidase (Selvaraj et al., 2015).

In our study, ergosine showed binding energies of −8.11 kcal/mol and −7.78 kcal/mol with the main protease and spike glycoprotein, respectively (Supplementary Tables S2 and S3). The grid-opening residues of the main protease active site that was involved in H bond formation are Met49, Asn142, and Gln189 (Figures 2E, F). This binding might interfere with the normal grid opening and prevent substrate binding to the main protease active site, which will ultimately reduce or completely block its activity. Met49 formed H bonds (H-donors) at distances of 3.67 with −0.3 energy. Asn 142 (H-acceptor) and GLN189 (pi-H) formed bonds with ergosine at distances of 2.81 and 4.05, with binding energies of −0.9 and −0.8, respectively. It interacted with the spike glycoprotein through four H bonds. The spike glycoprotein residues that are involved in H bond formation with ergosine are Gln98, Gly205, Lys562, and Gln102. Leu95 and Asn210 are the additional interactions shown in PyMOL (Figures 2G, H). PyMOL is a molecular visualization tool with a focus on 3D structure visualization, which can demonstrate various interaction details (Barber, 2021).

Gly205 formed a bond (H-donor) with ergosine at a distance of 3.02 with −3.3 energy. The other two residues, Lys562 and Gln102, formed an H bond (H-acceptor) with ergosine at a distance of 3.26 and 3.04 with −1.1 and 0.8 energy, respectively. It also made H-bonding interactions with Gln98 at a distance of 3.24.

From all the screened alkaloids of *R*. *apiculata* Bl. [Rhizophoraceae], ergosine exhibited the strongest binding affinity with target proteins. This plant has a number of therapeutic applications. It possesses anti-cholinesterase, antioxidant, antinociceptive, anti-plasmodia, anti-cancer, antimicrobial, antiemetic, and anti-diabetic activities. Its ethanolic extract can inhibit the growth of *Candida albicans*. It has tannins that possess inhibitory activity against notorious fungi (Baishya et al., 2020). This plant was also reported to have antiviral properties and showed an inhibitory effect on HIV disease (Huda et al., 2013; Nisar et al., 2019).

#### 3.3.3 Evodiamide C

**FIGURE 3:**
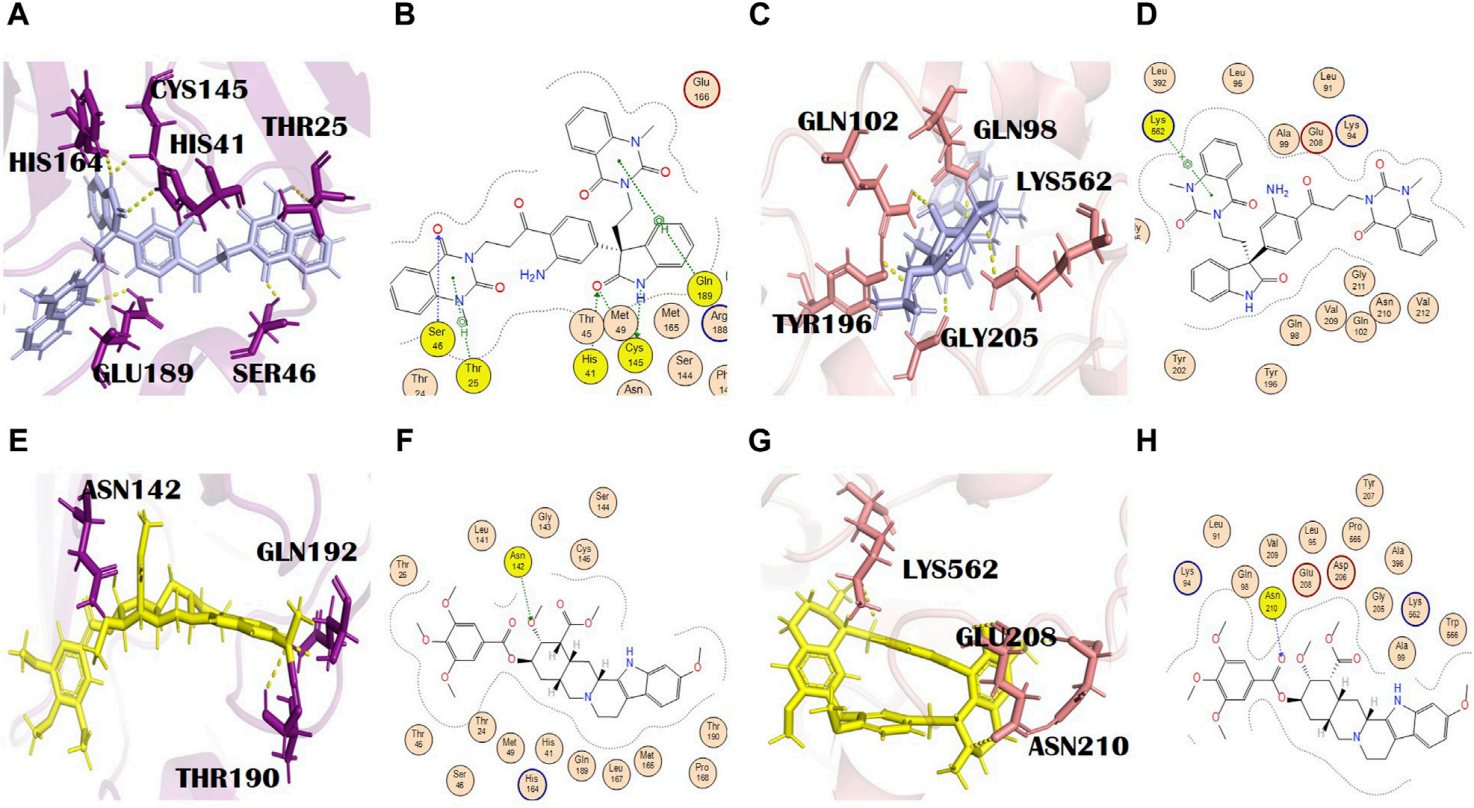
**(A)** 3D and **(B)** 2D molecular interactions of evodiamide C (light blue) with main protease residues (purple), **(C)** 3D and **(D)** 2D molecular interactions of evodiamide C (light blue) with spike glycoprotein residues (salmon), **(E)** 3D and **(F)** 2D molecular interactions of reserpine (yellow) with the main protease (purple), **(G)** 3D and **(H)** 2D molecular interactions of reserpine (yellow) with spike glycoprotein (salmon).

Evodiamide C belongs to the class of quinazoline alkaloids, is present in *E*. *rutaecarpa* (Juss.) Benth. [Rutaceae], and possesses antimicrobial activity (Su et al., 2018). The docking score of this metabolite with the main protease and spike glycoprotein was −9.15 and −9.05, respectively (Supplementary Tables S2 and S3). The main protease residues involved in H bonding with evodiamide C were Ser46, Thr25, and Gln189, and the residues of the catalytic dyad were Cys145 and His41. His164, with a bond distance of 2.2 Å, is the additional interaction shown in PyMOL (Figures 3A, B). This might result in complete inhibition of the main protease after binding with evodiamide C (Al-Rabia et al., 2021). Gln189 is involved in the formation of H bonds (pi-H) at a distance of 4.11 and an energy of 2.2. Similarly, Cys145 is involved in two H bond formations at distances of 3.25 and 3.48 with −1.5 and −1.4 energy, respectively. His41 is also involved in the formation of an H bond (H-acceptor) at a distance of 3.42 with −1.6 energy. Thr25 formed an H bond (pi-H) with −1.0 energy at a distance of 3.73. His164 is also involved in H bond formation with evodiamide C at a distance of 3.34. Evodiamide C interacted with Ser46 through the H bond at a distance of 3.29 with −1.9 energy. In the case of spike glycoproteins, evodiamide C was involved in the formation of one H bond (pi-cation) with Lys562 at a distance of 3.94 with −1.3 energy. In PyMOL results, it interacted with spike glycoprotein through five H bonds (Figures 3C, D). The residues included Gln98, Gln102, Thr196, Gly205, and Lys562, with bond distances of 1.8, 2.5, 2.4, 1.9, and 2.9, respectively.

*E*. *rutaecarpa* (Juss.) Benth. [Rutaceae] is an important part of traditional Chinese medicines, and in China, it is commonly known as Wu-Chu-Yu (Jiang and Hu, 2009). Different parts of this plant have various biological activities such as uterotonic, anti-adipogenic, antinociceptive, vasodilatory, thermoregulatory, anti-hypertensive, anti-inflammatory, and anti-obesity. It also possesses excellent anti-infective, anti-Alzheimer, and anti-cancer activities and therapeutic potential for the treatment of cardiovascular diseases. Most of the mentioned activities are attributed to alkaloids (Jiang and Hu, 2009; Liao et al., 2011; Tian et al., 2019). *E*. *rutaecarpa* (Juss.) Benth. [Rutaceae] also showed antiviral activity and effectively inhibited the influenza A virus (Dai et al., 2012). Out of the selected alkaloids of *E*. *rutaecarpa* (Juss.) Benth. [Rutaceae], evodiamide C displayed the strongest binding affinity with 6LU7 and 6LZG; hence, it needs further exploration for the treatment of SARS-CoV-2.

#### 3.3.4 Reserpine

Reserpine’s molecular formula and IUPAC name are C33H40N2O9 and methyl (1R,15S,17R,18R,19S,20S)-6,18-dimethoxy-17-(3,4,5-trimethoxybenzoyl) oxy-1,3,11,12,14,15,16,17,18,19,20,21-dodecahydroyohimban-19-carboxylate, respectively. It exhibited antibacterial and antioxidant properties (Begum et al., 2012). It belongs to the indole alkaloids isolated from the roots of *C*. *pareira* L. [Menispermaceae]. It also has anti-mutagenic and anti-cancer properties (Ramu et al., 2021). The docking scores of reserpine were −8.84 and −9.17 with 6LU7 and 6LZG, respectively (Supplementary Tables S2 and S3). The 6LU7 residue that was involved in the H bond formation (H-acceptor) with reserpine was Asn142. Furthermore, it showed two more interactions in PyMOL by residues Thr190 and Gln192 (Figures 3E, F). Asn142 formed an H bond with reserpine at a distance of 2.83 with −3.1 energy. This binding might slightly interfere with the active site rearrangement required for the substrate binding with the main protease. It formed three H bonds with three different residues of 6LZG (Figures 3G, H). Asn210 is involved in the formation of an H bond with reserpine at a distance of 2.95 with an energy of −3.1.

#### 3.3.5 Pelosine

**FIGURE 4:**
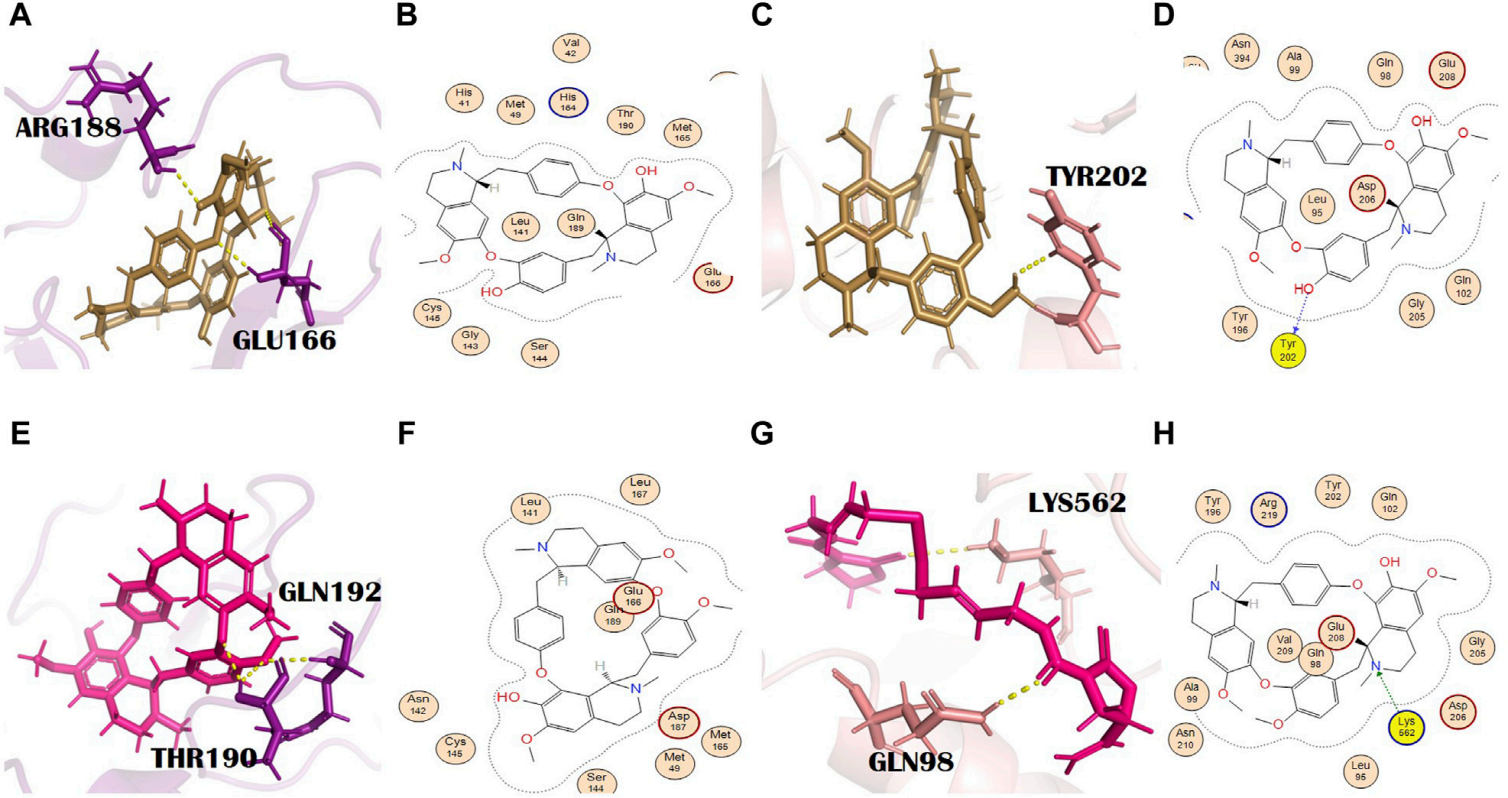
**(A)** 3D and **(B)** 2D molecular interactions of pelosine (sand) with main protease residues (purple), **(C)** 3D and **(D)** 2D molecular interactions of pelosine (sand) with spike glycoprotein residues (salmon), **(E)** 3D and **(F)** 2D molecular interactions of hayatinine (pink) with the main protease (purple), **(G)** 3D and **(H)** 2D molecular interactions of hayatinine (pink) with spike glycoprotein (salmon).

Pelosine, also known as berberine, is present in the root of *C*. *pareira* L. [Menispermaceae]. It belongs to the isoquinoline alkaloids. Its molecular formula and IUPAC name are C36H38N2O6 and (1S,16S)-10,25-dimethoxy-15,30-dimethyl-7,23-dioxa-15,30-diazaheptacyclo [22.6.2.23,6.18,12.118,22.027,31.016,34] hexatriaconta-3(36),4,6(35),8(34),9,11,18(33),19,21,24,26,31-dodecaene-9,21-diol, respectively. Pelosine showed docking scores of −7.93 and −7.77 with the main protease and spike glycoprotein, respectively (Supplementary Tables S2 and S3). It interacted with the main protease only through hydrophobic interactions with many significant residues, for example, the catalytically significant residues Cys145 and His41. Three H bond interactions were identified by PyMOL with residues Glu166 and Arg188. Glu166 formed two bonds with bond distances of 3.4 Å and 2.6 Å. Although the distance with Arg188 was 2.7 Å (Figures 4A, B), pelosine formed one H bond (H-donor type) with a spike glycoprotein residue (Tyr202) at a distance of 2.95 with an energy of −1.0 kcal/mol (Figures 4C, D). Among the top 12 dual alkaloids, Tyr202 was the one involved in the interaction with pelosine.

#### 3.3.6 Hayatinine

Hayatinine is isolated from the root of *C*. *pareira* L. [Menispermaceae] and belongs to the class of isoquinoline alkaloids. Its molecular formula and IUPAC name are C37H40N2O6 and (1S,16R)-10,21,25-trimethoxy-15,30-dimethyl-7,23-dioxa-15,30-diazaheptacyclo[22.6.2.23,6.18,12.118,22.027,31.016,34]hexatriaconta-3(36),4,6(35),8(34),9,11,18(33),19,21,24,26,31-dodecaen-9-ol, respectively. It has anti-plasmodial (Bhatt et al., 2020) and chemopreventive activities (Bala et al., 2017). The docking scores of hayatinine with the main protease and spike glycoprotein were −8.11 and −7.91, respectively (Supplementary Tables S2 and S3). Similar to pelosine, it interacted with the main protease only through hydrophobic interactions, yet these interactions involved many significant active site residues. However, three H bonds by residues Thr190 (two bonds) and Gln192 of the main protease are shown in PyMOL (Figures 4E, F). The bond distances with Thr190 were 2.2 Å and 2.4 Å, whereas the bond distance with Gln192 was 2.6 Å (Supplementary Table S2). Moreover, in the case of bonding with spike glycoprotein, it formed one H bond with spike protein. The Lys562 (spike glycoprotein residue) formed an H bond (pi-cation) at a distance of 3.24 with an energy of −1.3 (Figures 4G, H). Lys562 is found to be a common residue in the interaction profiles of the top 12 alkaloids. Gln98 is an additional interaction of hayatinine with spike glycoprotein that was observed in PyMOL with a bond distance of 2.1 Å (Supplementary Table S3).

#### 3.3.7 Homoaromoline

Homoaromoline is isolated from the root of *C*. *pareira* L. [Menispermaceae] (Rukachaisirikul et al., 2019). Its molecular formula and IUPAC name are C37H40N2O6 and (1R,14S)-6,20,25-trimethoxy-15,30-dimethyl-8,23-dioxa-15,30 diazaheptacyclo[22.6.2.29,12.13,7.114,18.027,31.022,33]hexatriaconta 3(36),4,6,9(35),10,12(34),18,20,22(33),24,26,31-dodecaen-21-ol, respectively. It had inhibitory potential against HSV-1 and HSV-2 (Sharma et al., 2022). It also showed a cytotoxic effect on colon cancer cells (Rukachaisirikul et al., 2019). Homoaromoline resulted in a docking score of −8.26 with the main protease (Supplementary Table S2). Homoaromoline formed an H bond (pi-H type) with the main protease through the Gln189 residue at a distance of 4.28 with −0.6 kcal/mol energy. His41, Glu166, and Gln189, with bond distances of 2.8 Å, 2.7 Å, and 2.4 Å, respectively, are the additional but significant residues that showed interactions with the main protease in PyMOL (Supplementary Figure S2A, B). In the case of spike glycoprotein, the docking score of homoaromoline was −7.88, and it formed an H bond (H-acceptor type) with spike glycoprotein (Supplementary Table S3). This H bond was formed with Lys562 at a distance of 3.05 with −1.4 energy. Five additional interactions with residues Gln98 (2.0 Å), His195 (2.4 Å), Asn210 (2.5 Å), and Arg219 (two bonds with distances of 2.3 and 2.7 Å) are identified in PyMOL (Supplementary Figure S2C, D).

The aforementioned alkaloids, namely, reserpine, (+)-homoaromoline, hayatinine, and pelosine, belong to *C*. *pareira* L. [Menispermaceae]. It is a popular medicinal plant in Ayurveda and has several pharmacologic activities (Bhatt et al., 2020). It also has antiviral potential. It is effectively used against the dengue virus (Sood et al., 2015; da Silva et al., 2020). Docking results indicated that this antiviral plant had great potential for anti-COVID-19 drug design.

#### 3.3.8 Isatithioetherin C

The docking scores of isatithioetherin C with the main protease and spike glycoprotein were −8.32 and −7.84, respectively (Supplementary Tables S2 and S3). Isatithioetherin C formed two H bonds with the main protease through Gln189 and Asn142 (Supplementary Figure S2E, F). Gln189 formed an H bond (H-acceptor type) at a distance of 3.08 with −0.7 energy. ASN142 exhibited an H bond (H-acceptor type) at a distance of 3.81 with −0.9 energy. Isatithioetherin C interacted with spike proteins through the formation of two H bonds with Gln98 and Lys562. Both of these bonds are H-acceptor types. Gln98 and Lys562 interact with isatithioetherin C at a distance of 3.06 and 4.03 with −1.5 and −6.9 energy, respectively. Interestingly, the same bonding pattern was observed with hayatinine in both cases. However, in the PyMOL binding pattern of isatithioetherin C with spike glycoprotein, four bonds were observed with Tyr202 (1.9 Å), Glu208 (two bonds with bond distances of 2.4 Å and 3.4 Å), and Asn210 (2.6 Å; Supplementary Figure S2G, H).

Isatithioetherin C is a sulfur-rich alkaloid present in the roots of *I. indigotica* Fort. [Brassicaceae]. This plant is used in Chinese traditional medicine. The roots and leaves of *I*. *indigotica* Fort. [Brassicaceae] are commonly named Ban-Lan-Gen and Da-Qing-Ye, respectively (Chen et al., 2021). It is an important plant with antiviral potential. It has an inhibitory effect on different types of viruses, including H1V1 virus, coronavirus, hepatitis B virus, anti-HSV-I, HSV-II, dengue virus II, cytomegalovirus, respiratory syncytial virus, cytomegalovirus, Newcastle virus, encephalitis B, mumps virus, and coxsackievirus (Chang et al., 2012; Yang et al., 2013; Lejars et al., 2020; Wang et al., 2020; Chen et al., 2021).

#### 3.3.9 N,alpha-L-rhamnopyranosyl vincosamide

**FIGURE 5:**
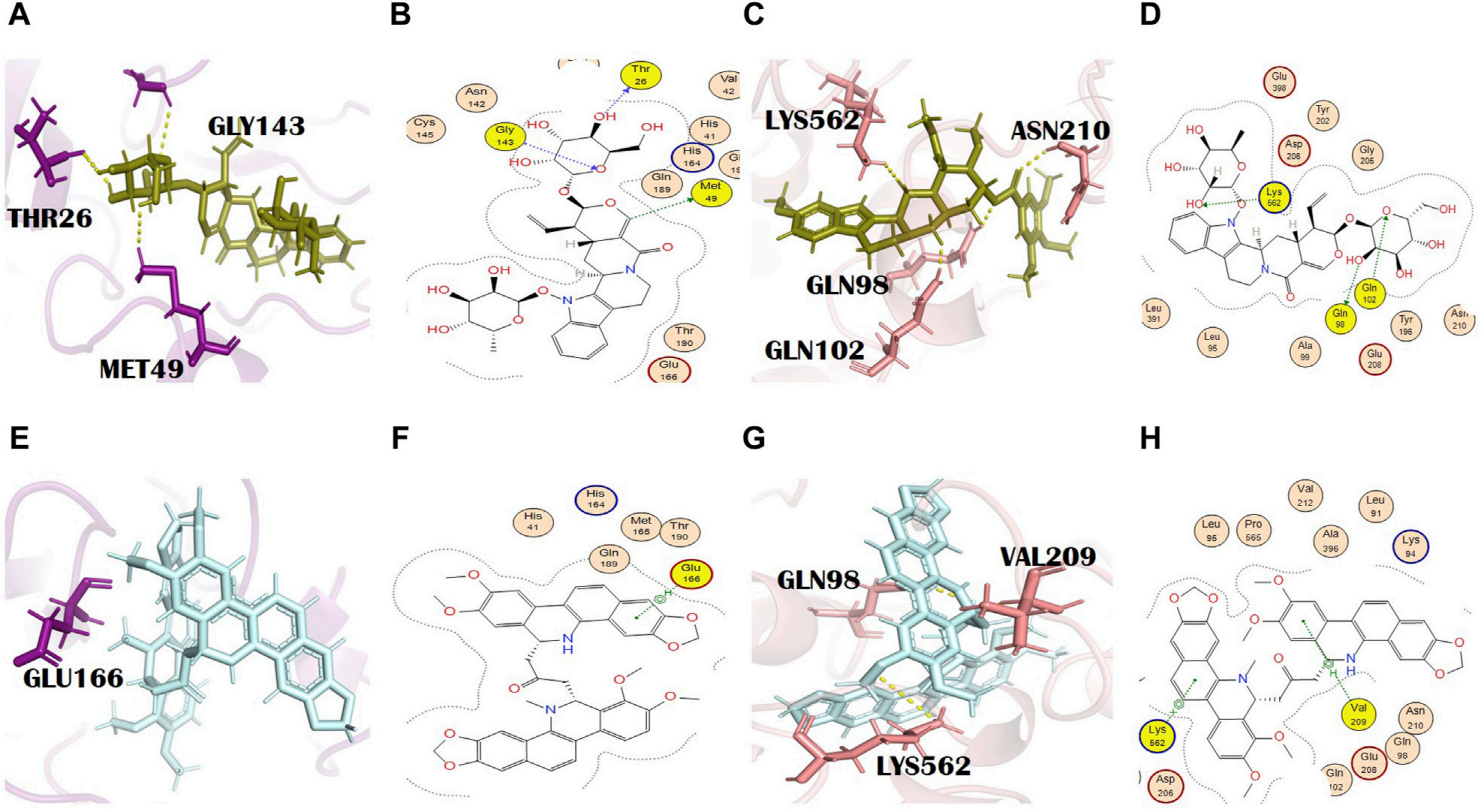
**(A)** 3D and **(B)** 2D molecular interactions of N,alpha-L-rhamnopyranosyl vincosamide (olive) with main protease residues (purple), **(C)** 3D and **(D)** 2D molecular interaction of N,alpha-L-rhamnopyranosyl vincosamide (olive) with spike glycoprotein residues (salmon), **(E)** 3D and **(F)** 2D molecular interactions of toddalidimerine (pale cyan) with the main protease (purple), **(G)** 3D and **(H)** 2D molecular interactions of toddalidimerine (pale cyan) with spike glycoprotein (salmon).

The docking scores of this metabolite against the main protease and spike glycoprotein were −8.82 and −8.67, respectively (Supplementary Tables S2 and S3). It formed three H bonds with the main protease residues. The residues involved in the formation of H bonds are Met49, Thr26, and Gly143 (Figures 5A, B). Thr26 and Gly143 formed H bonds at a distance of 3.11 and 3.05 with −0.8 and −1.7 energy, respectively. It formed three H bonds with the different residues of spike glycoprotein. The residues of spike glycoprotein involved in H bond formation with this ligand are Gln98 (H-donor), a common binding residue, Gln102 (H-acceptor), and Lys562 (H-acceptor). Asn210, with a bond distance of 2.2 Å, is another interaction shown in PyMOL (Figures 5C, D). Gln98, Gln102, and Lys562 formed H bonds at the distances of 2.66, 3.19, and 3.12 with energies of −1.3, −1.4, and −3.5, respectively. Interestingly, Gln98 is bound to almost all the lead alkaloids (Supplementary Tables S2 and S3). This residue might be easily accessible at the active site of spike glycoprotein and might play a key role in its substrate binding. N,alpha-L-rhamnopyranosyl vincosamide is an indole alkaloid isolated from the roots of *M*. *oleifera* Lam. [Moringaceae]. It also has antioxidant, anti-apoptotic, and anti-inflammatory activities (Alia et al., 2022). It shows a cardioprotective effect and thus decreases the chances of cardiovascular disease (Panda et al., 2013; Cheraghi et al., 2017). Our study confirmed the therapeutic potential of this alkaloid and suggested an agent that requires further experimentation. *M*. *oleifera* Lam. [Moringaceae] was traditionally used in Ayurvedic medicines. The pharmacologic activities of *M*. *oleifera* Lam. [Moringaceae] are anti-cancer, anti-inflammatory, antibacterial, anti-hypertensive, antimicrobial, antioxidant, antiviral, anti-allergic, radical scavenging, hepatoprotective, cardiovascular, and neuraceutical activities (Anzano et al., 2021). It has excellent inhibitory activity against herpes simplex type 1 virus (Kurokawa et al., 2016), HIV (Agoyi et al., 2014), hepatitis B virus (Anzano et al., 2021), and herpes zoster virus (Xiong et al., 2022).

#### 3.3.10 Toddalidimerine

Toddalidimerine is a dimeric benzophenanthridine alkaloid present in the roots of *T*. *asiatica* (L.) Lam. [Rutaceae]. This metabolite resulted in docking scores of −8.84 and −10.02 with the main protease and spike glycoprotein, respectively (Supplementary Tables S2 and S3). Toddalidimerine interacted with the main protease through its residue Glu166 and formed an H bond (pi-H type) at a distance of 4.67 and −0.6 energy (Figures 5E, F). It interacted with spike glycoproteins through two H bonds. Toddalidimerine formed two H bonds with Val209 and Lys562. Val209 (pi-H type) and Lys562 (pi-cation) interacted with toddalidimerine at distances of 4.49 and 3.34 with energies of −0.6 and −0.9, respectively. Gln98, with a bond distance of 2.6 Å, is an additional and significant interaction shown in PyMOL with spike glycoprotein (Figures 5G, H).

#### 3.3.11 Toddayanis

**FIGURE 6:**
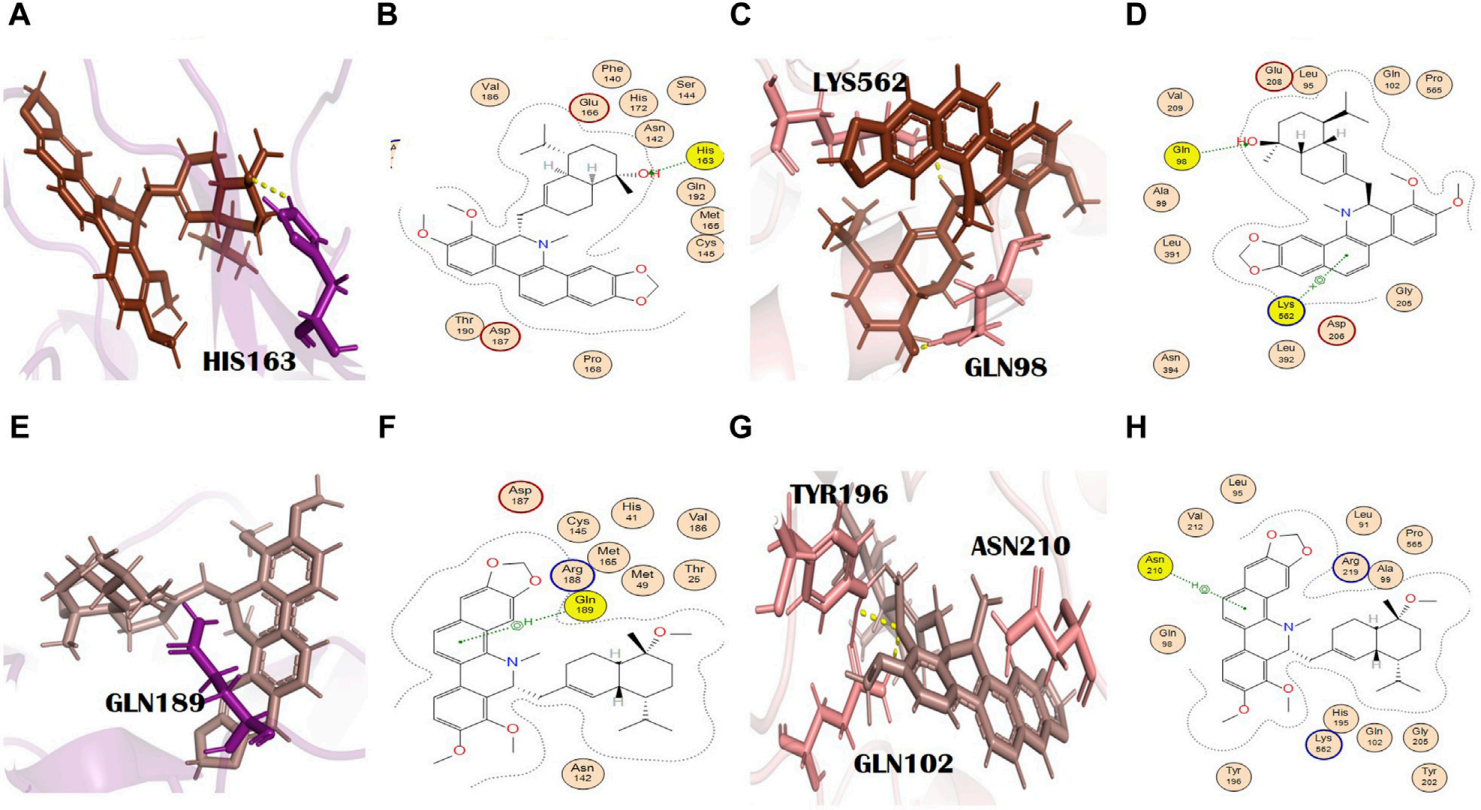
**(A)** 3D and **(B)** 2D molecular interactions of toddayanis (brown) with main protease residues (purple), **(C)** 3D and **(D)** 2D molecular interactions of toddayanis (brown) with spike glycoprotein residues (salmon), **(E)** 3D and **(F)** 2D molecular interactions of zanthocadinanine (dark salmon) with the main protease (purple), **(G)** 3D and **(H)** 2D molecular interactions of zanthocadinanine (dark salmon) with spike glycoprotein (salmon).

Toddayanis showed docking scores of −9.50 and −8.40 with the main protease and spike glycoprotein, respectively (Supplementary Tables S2 and S3). It formed one H bond with the main protease residues. His163 was involved in the formation of an H bond (H-acceptor type) with toddayanis at a distance of 2.91 with −0.9 energy (Figures 6A, B). It interacted with spike glycoprotein through two H bonds. The residues that were involved in the formation of the H bond with toddayanis were Gln98 and Lys562. Gln98 (H-acceptor type) and Lys562 (pi-cation type) formed H bonds at distances of 2.62 and 3.57 with −0.9 and −1.7 energy, respectively (Figures 6C, D). This binding pattern is common throughout the 12 alkaloids.

#### 3.3.12 Zanthocadinanine A

It is a benzo[c]phenanthridine alkaloid belonging to the isoquinoline alkaloids (Bisai et al., 2019). Zanthocadinanine A showed a docking score of −8.10 with the main protease (Supplementary Table S2). It interacted with a catalytically significant residue of the main protease, namely, Gln189, through the formation of an H bond (pi-H type) at a distance of 4.20 with −0.8 energy (Figures 6E, F). Zanthocadinanine A gave a docking score of −7.96 with spike glycoprotein (Supplementary Table S3). Zanthocadinanine formed one H bond (pi-H type) with Asn210 at a distance of 4.34 with −0.6 energy. Gln102 with a bond distance of 1.7 Å and Tyr196 with a bond distance of 2.6 Å are the residues that showed interaction in PyMOL (Figures 6G, H). Zanthocadinanine A, toddalidimerine, and toddayanis are medicinally important alkaloids present in *T*. *asiatica* (L.) Lam. [Rutaceae]. This plant is used in China and India. It is commonly known as the orange climber, da jiu jia, jian xue fei, and san bai bang (Alagaraj and Hematological, 2020; Zeng et al., 2021). Previously, it was reported for many pharmacologic activities and therapeutic implications, like anti-platelet aggregation activity; analgesic, anti-pyretic, anti-cancer, antimicrobial, and spasmolytic activities; wound healing; skin whitening; antioxidant and anti-malarial activities; cardiovascular protection; inhibition of Alzheimer disease pathogenesis; and bacteriostatic activity. *T*. *asiatica* (L.) Lam. [Rutaceae] has promising antiviral activity and inhibits HIV (Rashid et al., 2006; Priya et al., 2015) and the H1N1 influenza virus (Gakuubi et al., 2017). Besides, *T*. *asiatica* (L.) Lam. [Rutaceae] exhibited antibacterial and antifungal effects (Duraipandiyan et al., 2009).

In general, the results of the molecular interaction analysis of the top dual inhibiting alkaloids indicated a consistent interaction with the significant residues of the active sites of both target proteins, that is, Glu166, Gln189, Met49, Cys145, and Gln192 in the case of the main protease and Gln98, Asn210, and Lys562 in the case of the spike glycoprotein. The interaction profile of our top metabolites is like the standards of the main protease; however, it is different from the standard of spike glycoprotein. Notably, homoaromoline and evodiamide C interact particularly with the residues of the catalytic dyad in the S1 pocket of the main protease. It was previously established that the docking scores and therefore the binding affinity of the top dual inhibiting alkaloids are superior to the renowned standard inhibitors (N3, Paxlovid, and molnupiravir); additionally, the binding patterns indicate that most of these alkaloids bonded with a greater number of hydrogen bonds compared with the standards.

### 3.4 Docking validation

To check the credibility of our docking results, the docking validation step was performed for both target proteins. The RMSD values validate the docking process; the RMSD value lower than 2 Å confirms the accuracy of the docking protocol. For the main protease, a reference complex was prepared that was retrieved from the PDB (PDB ID: 6LU7). The second complex was prepared by docking the co-crystallized ligand, that is, N3 of 6LU7. The superimposition of these complexes was performed in the PyMOL software, which gave the final RMSD value of 0.725 Å, in which 284 atoms were perfectly aligned to 284 atoms (Figure 1C).

In the case of S-protein, the standard compound (molnupiravir) was not actually the co-crystallized ligand, and so the two complexes, the first of the docked complex of the standard and the second of the re-docked complex of the standard, were prepared and superimposed in the PyMOL software (Figure 1F). All the 791 atoms were exclusively aligned without any rejection, with an RMSD value of 0.000 Å and a match align score of 4301.000. This result shows the excellent docking protocol and attests to the accuracy of our results.

### 3.5 Simulation trajectory analysis

Based on the computational docking scores and subsequent interactions observed within the binding pocket residues, the top three pharmaceutical metabolites, namely, adouetine Y, ergosine, and evodiamide C, were subjected to MD simulations for a duration of 100 ns to validate their binding stability with the proteins 6LU7 and 6LZG. Furthermore, to validate the binding mechanism of selected alkaloids and gain a more comprehensive understanding of interaction patterns, the reference drugs as well as apoproteins were analyzed using MD. As performed previously (Shah et al., 2024), the evaluation of complex stability was based on comprehensive analyses, including RMSD and RMSF assessments. RoG and SASA analyses were also performed to evaluate the conformational changes throughout the simulation run.

**FIGURE 7:**
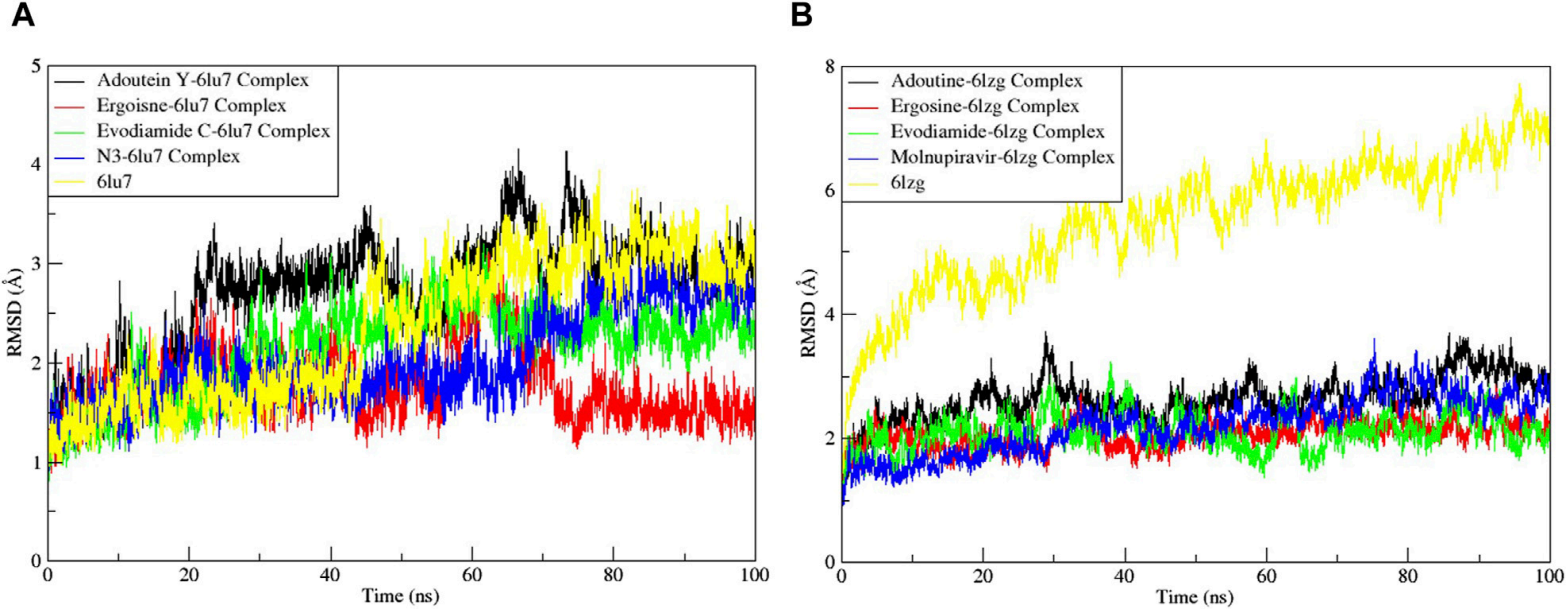
Root mean square deviation (RMSD) of MD simulation trajectory analysis of the complexes of three ligands, with respective standards of proteins and apoproteins, **(A)** main protease (6LU7) and **(B)** spike glycoprotein (6LZG). The variation in the RMSD between the α-C atoms of proteins and the top three ligands. The *y*-axis shows the protein RMSD shifts, whereas the *x*-axis shows the course of time.

**FIGURE 8:**
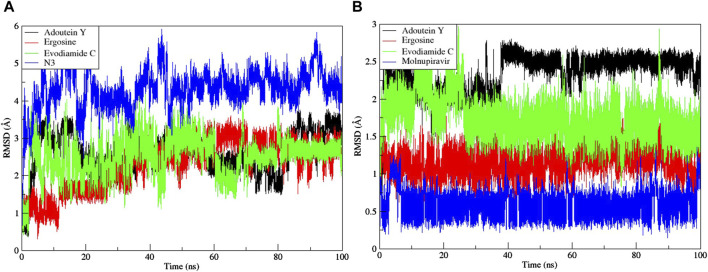
Root mean square deviation (RMSD) of MD simulation trajectory analysis of the three ligands and respective protein standards with **(A)** main protease (6LU7) and **(B)** spike glycoprotein (6LZG). The *y*-axis shows the protein RMSD shifts, whereas the *x*-axis shows the course of time.

Noteworthy observations for protein 6LU7 were revealed by RMSD analyses. The overall deviation of ligands within the binding pocket of the target protein was assessed. The root mean square deviation (RMSD) provided insight into the structural integrity of complexes and apoproteins over 100 ns (Figures 7, 8). The adouetine Y complex bound to 6LU7 exhibited slightly more pronounced structural deviations around 68 and 74 ns compared with its counterparts in other 6LU7 complexes. Though the adouetine Y complex showed minor overall perturbations due to ligand interaction, a detailed analysis of RMSD across all complexes revealed that they showed a similar trend, like the reference drug N3 (RMSD, 2.05 Å). They also achieved stability with RMSD values around 2 Å–3 Å toward the end of the 100-ns period. Additionally, it is evident from the RMSD graph of apoprotein that the binding of ergosine and evodiamide C induced stability to the overall structure of the protein (Figure 7A). In the RMSD analysis of the three ligands bound with 6LU7, evodiamide C was found to be less stable in comparison with the other two ligands, whereas N3 (the reference drug) exhibited a more distorted pattern with an average RMSD of 4.31 Å (Figure 8A). Moreover, the fluctuations were constant for ligand complexes with 6LZG at the RMSD values of 1 Å, 2 Å, and 2.5 Å, respectively, for ergosine, evodiamide C, and adouetine Y. Similar to the studied metabolites, the reference drug molnupiravir also stabilized earlier with an average RMSD of 2.21 Å, whereas the apoprotein did not show much stable behavior in comparison with docked complexes (Figure 7B). Similarly, the adouetine Y and evodiamide C complexes with 6LZG displayed a bit more significant structural disorientation at 30 ns and around 90 ns compared with the 6LZG complex with ergosine. Moreover, all three complexes gain stability around 2.5 Å at the end of the 100-ns period. All ligand–protein complexes, including the selected ligands and reference drug molnupiravir, at the initial and endpoint (0 ns and 100 ns) of the simulation indicated minimal structural variations, with the ligands maintaining their positions within the 6LZG binding site (Figure 8B). Moreover, after conducting our analysis, it is evident that the three alkaloids exhibit a comparable ability to bind and block the active site residues as the reference drugs (molnupiravir and N3).

**FIGURE 9:**
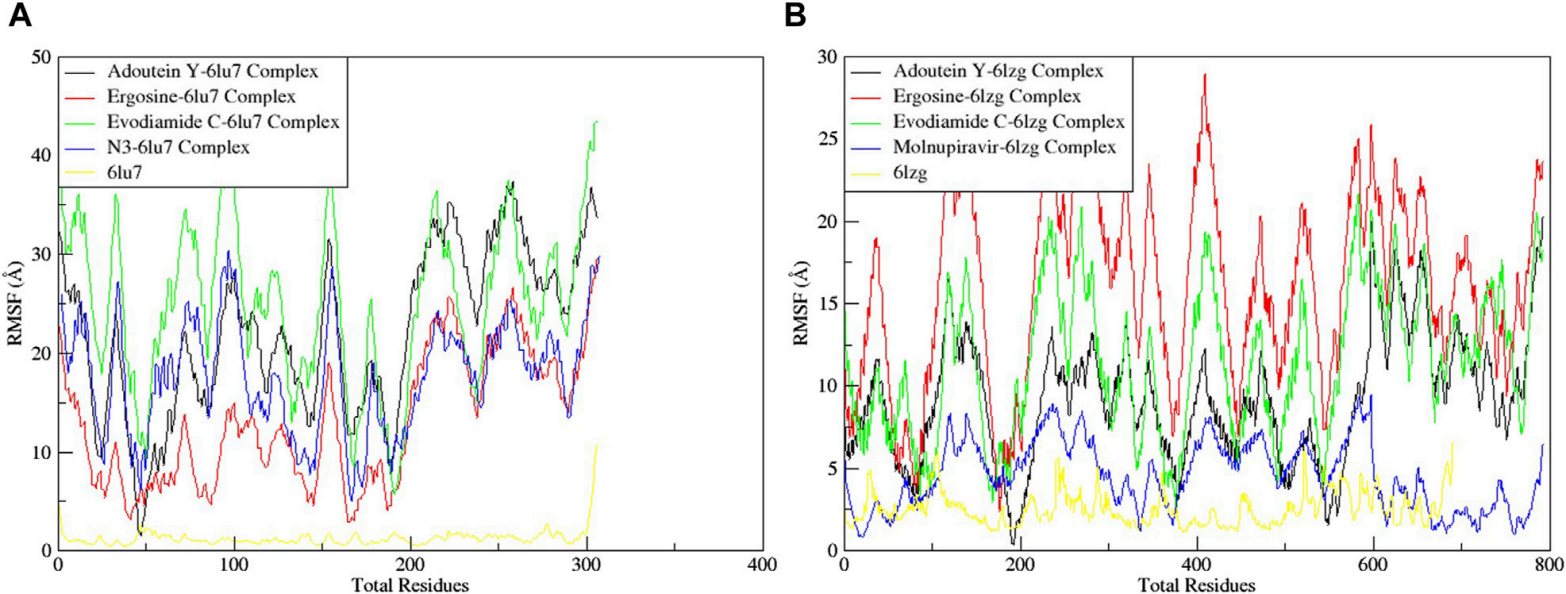
Root mean square fluctuation (RMSF) of MD simulation trajectory analysis of the **(A)** main protease (6LU7) and **(B)** spike glycoprotein (6LZG) residues complexed with the selected top three ligands and respective protein standards. The *y*-axis shows the protein RMSF shifts, whereas the *x*-axis shows the number of residues.

**FIGURE 10:**
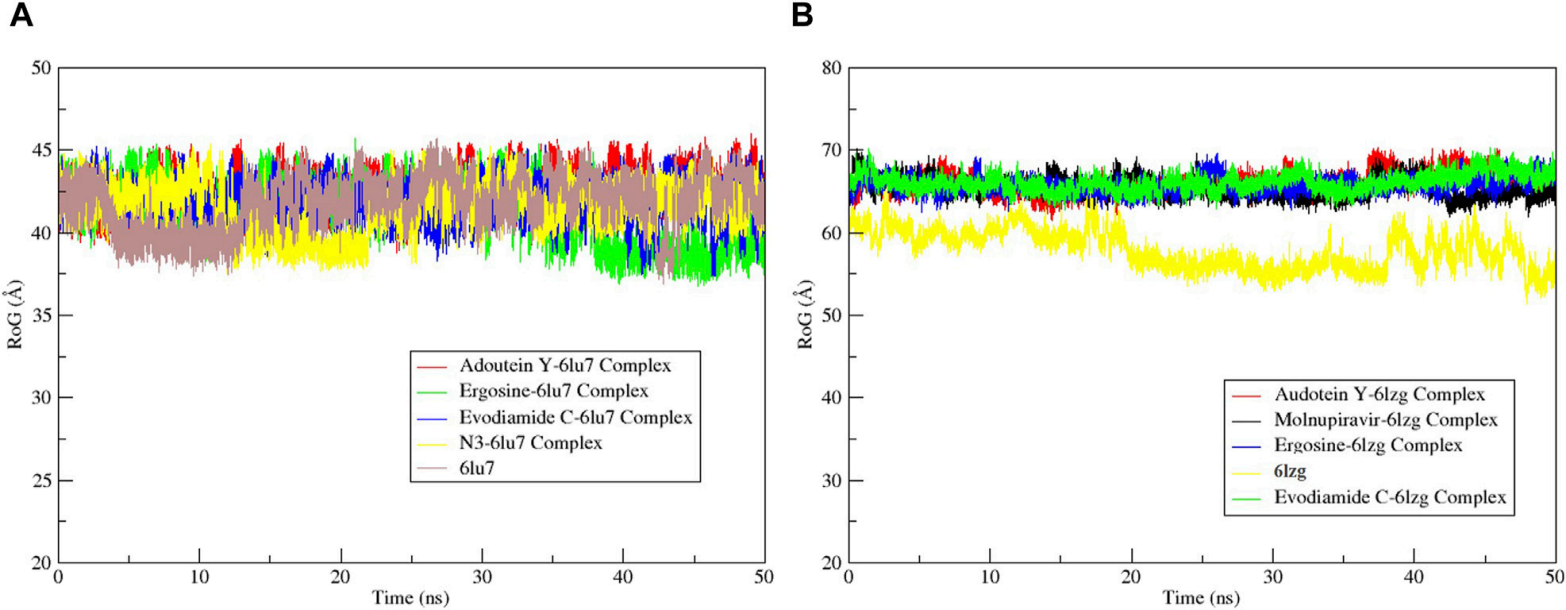
Radius of gyration (RoG) calculation of MD simulation trajectory analysis of the **(A)** main protease (6LU7) and **(B)** spike glycoprotein (6LZG) residues complexed with the selected top three ligands, respective protein standards, and apoproteins. The *y*-axis shows the protein RoG values, whereas the *x*-axis shows the course of time.

**FIGURE 11:**
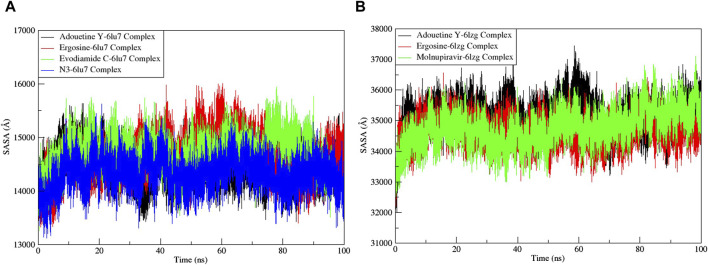
Solvent accessible surface area (SASA) analysis of the MD simulation trajectory analysis of the complexes of three ligands and respective protein standards. **(A)** Main protease (6LU7) and **(B)** spike glycoprotein (6LZG). The *y*-axis shows the protein SASA values, whereas the *x*-axis shows the course of time.

The RMSF served as a measure of atomic mobility, delineating the flexible and rigid segments within the drug–target complexes. Furthermore, the RMSF analyses indicated similar patterns for the protein 6LU7 when bound to different ligands. The corresponding graph demonstrated a generally stable formation in the complexes involving adouetine Y, ergosine, evodiamide C, and N3 (Figure 9A). The fluctuations across the 6LZG complexes with adouetine Y, ergosine, evodiamide C, and molnupiravir were notably variable and consistent, with minor deviations primarily at the terminal regions (Figure 9B). It was observed that the average RMSF of the apoproteins (6LU7 and 6LZG) was lower than that of the docked complexes. Docked complexes exhibit a multitude of fluctuations, predominantly attributable to the formation of unique bonds and interactions that are necessary for the formation of stable complexes. The root mean square distance of an object’s components from its center of gravity or a specified axis is denoted by the radius of gyration. With the aid of this analysis, the compactness of a protein subjected to dynamic forces can be determined. Elevated RoG values indicate a reduced degree of rigidity and enhanced mobility. Pre- and post-binding to specific ligands and reference drugs, the radius of gyration of C-α atoms in apoproteins was quantified. For Apo 6LU7, the average RoG value was 41.8 Å, whereas for docked complexes Apo plus adouetine Y, ergosine, evodiamide C, and N3, the average values were 42.6 Å, 41.1 Å, 41.9 Å, and 41.6 Å, respectively (Figure 10 A). Conversely, the average RoG of Apo 6LZG, adouetine Y, ergosine, evodiamide C, and molnupiravir docked with 6LZG systems were 57.9 Å, 66.0 Å, 65.8 Å, 66.1 Å, and 65.4 Å, respectively (Figure 10B), which implies that all the systems showed very similar compactness. SASA is a metric used to quantify the surface area that is permeable to a given solvent. Protein–ligand binding affinity is significantly influenced by the SASA of the ligand-binding site. It is an additional significant thermodynamic stability metric that evaluates protein folding and surface area changes throughout a simulation; greater SASA values indicate a protein volume increase. A comparable result to the one reported by RoG indicates that the complex plots exhibited reduced SASA fluctuations over the course of the 100-ns simulation period. This suggests that adouetine Y, ergosine, evodiamide C, and reference drugs had a lesser effect on reducing the surface area of 6LU7 and 6LZG (Figure 11A, B). The SASA results of this investigation corroborated the RoG results about the greater stability of 6LU7 and 6LZG in their binding to the top three alkaloids and reference drugs, thereby providing additional evidence for the stability of complexes.

MD simulations of 100 ns for the protein 6LZG complexed with three different metabolites demonstrated the attainment of stability. This pattern suggests effective binding between the protein and the ligands. This showcased the movement of certain residues, where only those in the protein’s flexible areas exhibited noticeable fluctuations. Due to the absence of significant disturbances in the complex, stability was observed across all three complexes.

These findings collectively underscore the superior stability and binding affinity of adouetine Y, ergosine, and evodiamide C complexes with 6LU7 and 6LZG, positioning them as promising candidates for further investigation and potential therapeutic applications.

### 3.6 Binding free energy determination

**FIGURE 12:**
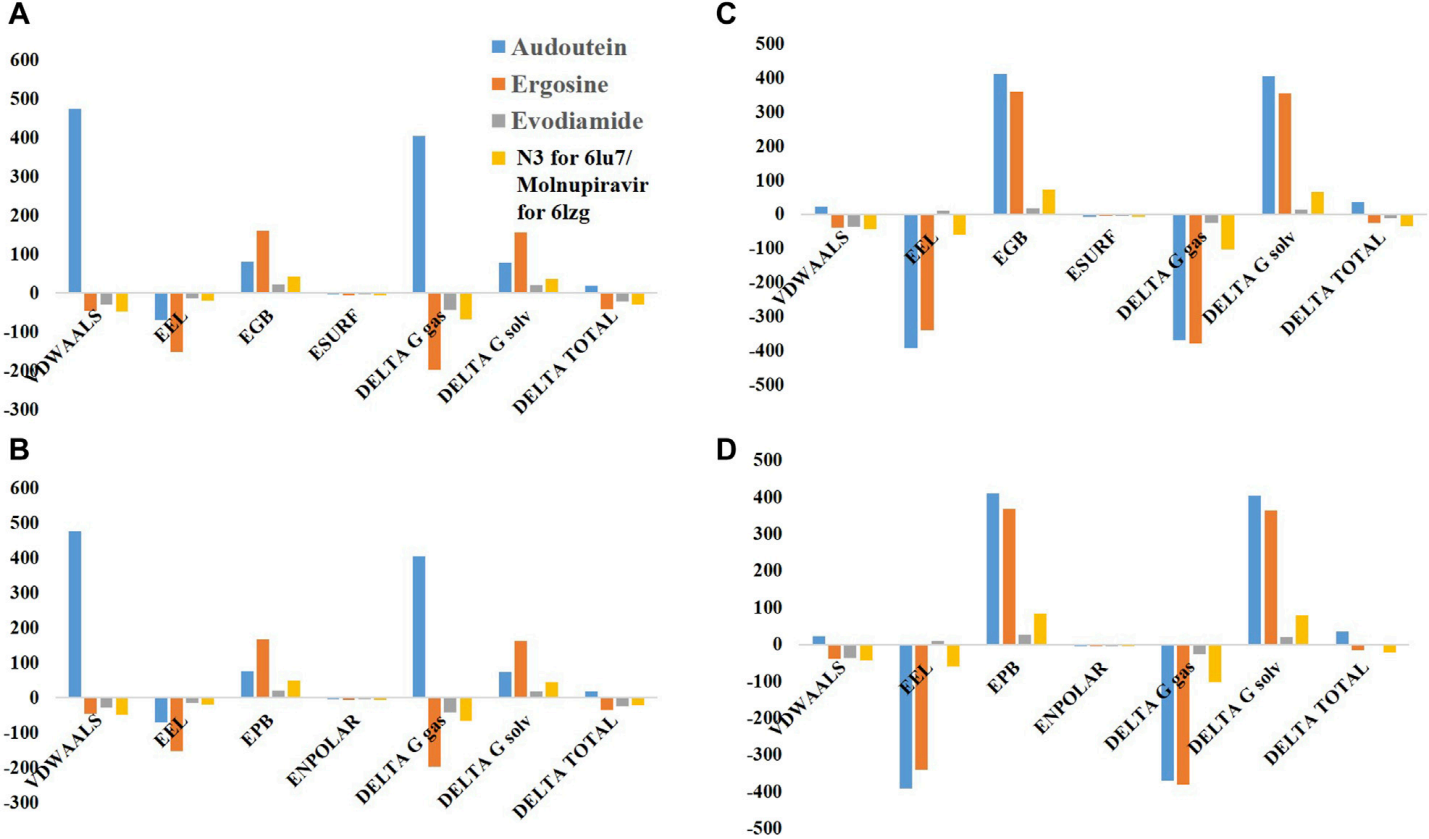
Comparative binding free energy calculations for SARS-CoV-2 protease structures using MM-PBSA (Poisson–Boltzmann model solvation energy) and MM-GBSA (generalized Born model solvation energy) techniques. **(A)** MM-GBSA for 6LU7, **(B)** MM-PBSA for 6LU7, **(C)** MM-GBSA for 6LZG, and **(D)** MM-PBSA for 6LZG.

For the SARS-CoV-2 proteins (6LU7 and 6LZG) and their three inhibitors (adouetine, ergosine, and evodiamide), as well as for their respective standards (N3 and molnupiravir), our simulations using the MM-GBSA and MM-PBSA methods provided key findings on how well they bind and their energy dynamics (Figure 12).

In the MM-GBSA model for 6LU7, the van der Waals (VDWAALS) contributions for adouetine are notably high, indicating strong hydrophobic interactions, whereas ergosine and evodiamide suggest weaker hydrophobic interactions. Electrostatic interactions (EEL) are unfavorable across all inhibitors, with ergosine exhibiting the most unfavorable electrostatics. The solvation energy component (EGB) and surface energy term (ESURF) further influence the binding energetics, resulting in a positive delta total for adouetine, which suggests further investigation. Ergosine and evodiamide show delta total values of −41.09 kcal/mol and −21.66 kcal/mol, respectively, which are more aligned with expectations for favorable binding interactions (Figure 12A).

In the MM-PBSA model for 6LU7, the VDWAALS and EEL components follow the same trend as in the MM-GBSA model. However, the polar solvation energy (EPB) and nonpolar solvation energy (ENPOLAR) contribute to the solvation free energy (DELTA G solv), leading to slightly more favorable delta total values of −34.08 kcal/mol and −24.00 kcal/mol for ergosine and evodiamide, respectively, indicating more stable interactions in this model (Figure 12B). In comparison with N3, the VDWALLS, ESURF, and ENPOLAR values of these three inhibitors were comparable; however, other parameters were potentially higher for adouetine and ergosine. Conversely, the delta total values of ergosine and evodiamide were found to be similar to those of N3, indicating that both ergosine and evodiamide could potentially mimic or compete with N3 in their biological activities, possibly involving similar binding sites or mechanisms of action (Figures 12A, B).

For the MM-GBSA model of 6LZG, adouetine demonstrates weak VDWAALS energy and highly unfavorable EELs, yet it shows a nonspontaneous or less favorable binding interaction with the overall delta total of 37.5438 kcal/mol. Ergosine shows negative VDWAALS and EEL energies, suggesting some favorable interactions. However, its positive EGB leads to a negative delta total of −24.84 kcal/mol, indicative of a favorable binding interaction. Moreover, the results of both the VDWAALS and EEL energies of evodiamide, with a slightly positive EGB, suggest a favorable but relatively weak binding interaction compared with ergosine. Its delta total is −10.49 kcal/mol (Figure 12C).

The MM-PBSA model of 6LZG for adouetine indicates that the VDWAALS and EEL components are similar to the MM-GBSA model, but the EPB is slightly lower. The delta total remains positive at 35.2 kcal/mol, indicating a less favorable binding interaction. Ergosine shows a total binding energy of −16.06 kcal/mol in the PB model, slightly less than in the GB model, still indicating a favorable binding interaction. Lastly, evodiamide has a total binding energy value of −3.52 kcal/mol in the PB model, which is less negative than the GB model, suggesting a weaker but favorable binding interaction (Figure 12D). In comparison with molnupiravir for 6LZG, the VDWAALS, ESURF, and ENPOLAR values of both ergosine and evodiamide are similar to the results with the standard N3 for the 6LU7 protein. However, the other energies are potentially higher than those of molnupiravir in the case of adouetine and ergosine and potentially lower in the case of evodiamide. Overall, the delta total of ergosine is comparable with that of molnupiravir. This suggests that ergosine might exhibit similar efficacy or binding characteristics when targeting the 6LZG protein (Figures 12C, D).

Conclusively, in both models for both proteins, ergosine and evodiamide exhibit favorable binding interactions, with ergosine showing a relatively stronger binding affinity. On the contrary, the positive total binding energy for adouetine suggests a nonspontaneous binding profile. Moreover, ergosine might exhibit similar efficacy or binding characteristics to both standards when targeting both 6LU7 and 6LZG proteins, raising the possibility of its use as an alternative therapeutic option. The differences in delta total values between GB and PB for each inhibitor highlight the influence and sensitivity of solvation parameters in the applied models.

### 3.7 Bioactivity score

The 12 lead alkaloids that showed dual inhibitory interactions with the receptor proteins were subjected to bioactivity score analysis through the Molinspiration tool. Based on the findings of this tool, biologically active compounds have a bioactivity score of >0.0, moderately active compounds have a bioactivity score between −0.50 and 0.0, and compounds are considered inactive if their bioactivity score value is <−0.50 (Veber et al., 2002). The results of the bioactivity score analysis indicated that 10 lead alkaloids showed promising bioactivity scores. Evodiamide C and toddalidimerine do not show a good bioactivity score. These results clearly indicated that adouetine Y, ergosine, and isatithioetherin C were better drug candidates based on their bioactivity score analysis (Table 1).

**TABLE 1:** Bioactivity score of lead alkaloids with dual inhibitor potential.

Sr. No.	Compound name	GPCR ligand	Ion channel modulator	Kinase inhibitor	Nuclear receptor ligand	Protease inhibitor	Enzyme inhibitor
*	Remdesivir	0.27	−0.35	0.20	−0.48	0.49	0.3
1	Adouetine Y	0.08	−0.48	−0.26	−0.41	0.36	−0.10
2	Ergosine	0.88	−0.04	−0.18	−0.27	0.29	0.05
3	Evodiamide C	−0.61	−1.55	−1.18	−1.27	−0.61	−0.86
4	Hayatinine	−0.02	−0.61	−0.49	−0.54	−0.07	−0.30
5	Homoaromoline	−0.02	−0.61	−0.49	−0.54	−0.07	−0.30
6	Isatithioetherin C	0.23	−0.10	−0.23	0.11	0.37	0.34
7	N,alpha-L-rhamnopyranosyl vincosamide	−0.21	−0.82	−0.72	−0.91	−0.10	−0.17
8	Pelosine	0.05	−0.48	−0.40	−0.44	−0.04	−0.21
9	Reserpine	0.10	−0.36	−0.39	−0.36	−0.02	−0.21
10	Toddalidimerine	−1.40	−2.50	−2.06	−2.12	−1.14	−1.89
11	Toddayanis	0.12	−0.41	−0.39	0.10	−0.21	−0.08
12	Zanthocadinanine A	0.07	−0.53	−0.41	0.04	0.20	−0.13

### 3.8 Drug-like ability

Drug-likeness and physiochemical properties are closely associated with each other. Drug-likeness analysis in Swiss ADME online software comprises five rules: Lipinski’s rule, Ghose’s rule, Veber’s rule, Egan’s rule, and Muegge’s rule. If the proposed compound is unable to follow two or more rules, then it might be considered orally inactive. Drug-likeness was mainly based on Pfizer’s or Lipinski’s rule of five (Benet et al., 2016). Adouetine Y follows the Lipinski rule (one violation: MW > 500). It also follows the Veber and Egan rules of drug-likeness. Ergosine, hayatinine, pelosine, and homoaromoline follow the Lipinski rule with one violation (MW > 500). Like adouetine Y, these three metabolites also follow the Veber and Egan rules. Isatithioetherin C only followed the Lipinski rule with one violation (MW > 500) but did not follow the remaining rules of drug-likeness (Table 2). Reserpine did not follow Lipinski’s rule, but it obeyed the Veber and Egan rule of drug-likeness. Toddalidimerine, zanthocadinanine A, and toddayanis followed the Veber drug-likeness rule, but they did not complete the criteria to follow the Lipinski rule or the Egan rule due to two violations (Supplementary Table S4; two violations: MW > 500, NorO > 10). Evodiamide C was the only metabolite that did not follow any rule of drug-likeness.

**TABLE 2:** Drug-likeness evaluation of dual-active alkaloids.

Sr. No.	Name	Lipinski’s rule	Veber	Egan	Bioavailability score
*	Remdesivir	-	-	-	-
1	Adouetine	Yes	Yes	Yes	0.55
2	Ergosine	Yes	Yes	Yes	0.55
3	Evodiamide C	No	No	No	0.17
4	Hayatinine	Yes	Yes	Yes	0.55
5	Homoaromoline	Yes	Yes	Yes	0.55
6	Isatithioetherin C	Yes	No	No	0.55
7	N,alpha-L-rhamnopyranosyl vincosamide	No	No	No	0.11
8	Pelosine	Yes	Yes	Yes	0.55
9	Reserpine	No	Yes	Yes	0.17
10	Toddalidimerine	No	Yes	No	0.17
11	Toddayanis	No	Yes	No	0.17
12	Zanthocadinanine A	No	Yes	No	0

Standard compounds did not have the required properties to meet the criteria of drug-likeness. The bioavailability score of evodiamide C, reserpine, toddalidimerine, toddayanis, and zanthocadinanine was 0.17, and N,alpha-L-rhamnopyranosyl vincosamide had a bioavailability score of 0.11 (Table 2). All the remaining metabolites had a bioavailability score of 0.55. In summary, approximately all of the metabolite’s bioavailability scores are comparable with or higher than the standard (Table 2).

### 3.9 ADMET analysis

ADMET analysis plays a central role in drug design before the clinical trial. Early ADMET analysis using software facilitates a decrease in drug labor and cost during a clinical trial (Pires et al., 2015). The ADMET analysis of lead metabolites was carried out through pkCSM online software. The detailed description of the absorption, distribution, metabolism, excretion, and toxicity of these six selected metabolites was as follows.

#### 3.9.1 Absorption parameter analysis

Skin permeability, aqueous solubility, gastrointestinal (GI) absorption, Caco-2 permeability, P-glycoprotein substrate, and P-glycoprotein inhibitor are important parameters that affect the absorption of the drug (Dahlgren and Lennernäs, 2019). The standard, remdesivir, showed −3.07 log mol/L of water solubility. Four out of the 12 selected metabolites showed higher water solubility than the standard. These four metabolites, hayatinine, N,alpha-L-rhamnopyranosyl vincosamide, pelosine, and toddalidimerine, showed more water solubility than standard. The results indicated that N,alpha-L-rhamnopyranosyl vincosamide showed the highest ability to dissolve in water. All the remaining metabolites showed less solubility than the standard, and zanthocadinanine showed the least capacity to be soluble in water (−6.109 log mol/L; Table 3). The second and third important parameters that affect drug absorption are GI absorption and Caco-2 permeability, respectively. The bioavailability of the drug depends upon GI absorption and Caco-2 permeability. A compound that has GI absorption >30% shows better absorbance in the body. All the leads showed excellent GI absorption. Their GI absorption is higher than the standard, except for N,alpha-L-rhamnopyranosyl vincosamide. Reserpine and toddalidimerine are 100% absorbed in the body through GI absorption (Table 3).

**TABLE 3:** ADMET properties of dual-active alkaloids.

Sr. No.	Property	Model name	*	1	2	3	4	5	6	7	8	9	10	11	12	Unit
1	Absorption	Water solubility	−3.07	−3.6	−3.5	−3.1	−3.0	−3.9	−4.4	−2.8	−2.9	−5.3	−2.9	−5.5	−6.1	Numeric (log mol/L)
		Caco-2 permeability	0.6	0.5	0.6	0.1	0.4	0.4	0.8	0.6	0.6	1.0	0.3	1.0	0.9	Numeric (log Papp in 10–6 cm/s)
		Intestinal absorption	71.1	87.1	75.5	92.8	91.2	90.0	86.2	33.0	91.6	100	100	96.5	98.5	Numeric (% absorbed)
		Skin permeability	−2.7	−2.7	−2.7	−2.7	2.7	−2.7	−2.8	−2.7	−2.7	−2.7	−2.7	−2.7	−2.7	Numeric (log Kp)
		P-glycoprotein substrate	Yes	Yes	Yes	No	Yes	Yes	Yes	Yes	Yes	Yes	No	Yes	No	Categorical (yes/no)
		P-glycoprotein I inhibitor	Yes	Yes	Yes	Yes	Yes	Yes	No	Yes	Yes	Yes	Yes	Yes	Yes	Categorical (yes/no)
		P-glycoprotein II inhibitor	No	Yes	Yes	Yes	Yes	Yes	No	No	Yes	Yes	Yes	Yes	Yes	Categorical (yes/no)
2	Distribution	VDss (human)	0.3	−0.0	1.1	−1.4	−0.4	−0.6	−0.6	−0.0	−0.3	0.3	−1.0	0.0	0.5	Numeric (log L/kg)
		Fraction unbound (human)	0.0	0.1	0.2	0.3	0.3	0.2	0.2	0.3	0.2	0.0	0.4	0.1	0.1	Numeric (Fu)
		BBB permeability	−2.0	−0.4	−0.4	−0.6	−0.8	−0.9	−1.6	−1.3	−0.7	−0.7	−0.8	−0.1	−0.4	Numeric (log BB)
		CNS permeability	−4.6	−2.4	−2.5	−3.4	−2.3	−2.3	−3.2	−4.5	−2.3	−3.3	−2.8	−1.2	−1.3	Numeric (log PS)
3	Metabolism	CYP2D6 substrate	No	No	No	No	No	No	No	No	No	No	No	No	No	Categorical (yes/no)
		CYP3A4 substrate	Yes	Yes	Yes	Yes	Yes	Yes	Yes	No	Yes	Yes	Yes	Yes	Yes	Categorical (yes/no)
		CYP1A2 inhibitor	No	No	No	No	No	No	No	No	No	No	No	No	No	Categorical (yes/no)
		CYP2C19 inhibitor	No	Yes	No	Yes	No	Yes	No	No	No	No	No	No	No	Categorical (yes/no)
		CYP2C9 inhibitor	No	Yes	No	Yes	No	No	No	No	No	No	No	No	No	Categorical (yes/no)
		CYP2D6 inhibitor	No	No	No	No	No	No	No	No	No	No	No	No	No	Categorical (yes/no)
		CYP3A4 inhibitor	No	Yes	Yes	Yes	No	No	No	No	No	Yes	No	No	No	Categorical (yes/no)
4	Excretion	Total clearance	0.1	0.3	0.6	0.7	0.6	0.7	0.3	0.2	0.6	0.5	0.3	−0.5	−0.4	Numeric (log mL/min/kg)
		Renal OCT2 substrate	No	No	No	No	Yes	No	No	No	Yes	No	No	No	No	Categorical (yes/no)
5	Toxicity	AMES toxicity	No	No	No	No	Yes	Yes	No	No	Yes	No	No	No	No	Categorical (yes/no)
		Maximum tolerated dose (human)	0.1	−0.5	−1.1	0.3	0.2	0.2	2.6	−0.6	0.3	−0.1	0.4	0.2	0.0	Numeric (log mg/kg/day)
		hERG I inhibitor	No	No	No	No	No	No	No	No	No	No	No	No	No	Categorical (yes/no)
		hERG II inhibitor	Yes	Yes	Yes	Yes	Yes	Yes	No	Yes	Yes	Yes	Yes	Yes	Yes	Categorical (yes/no)
		Oral rat acute toxicity (LD50)	2.0	2.7	3.2	2.9	2.5	2.5	2.6	2.7	2.5	2.9	2.6	2.9	3.2	Numeric (mol/kg)
		Oral rat chronic toxicity	1.6	2.6	2.5	1.8	1.1	1.2	1.1	3.4	1.2	1.1	−0.9	0.9	0.9	Numeric (log mg/kg_bw/day)
		Hepatotoxicity	Yes	Yes	No	Yes	No	No	Yes	No	No	No	No	No	No	Categorical (yes/no)
		Skin sensitivity	No	No	No	No	No	No	No	No	No	No	No	No	No	Categorical (yes/no)
		*Tetrahymena pyriformis* toxicity	0.2	0.2	0.2	0.2	0.2	0.2	0.3	0.2	0.2	0.2	0.2	0.2	0.2	Numeric (log µg/L)

^a^ 1, adouetine Y); 2, ergosine; 3, evodiamide C; 4, hayatinine; 5, homoaromoline; 6, isatithioetherin C; 7, N,alpha-L-rhamnopyranosyl vincosamide; 8, pelosine; 9, reserpine; 10, toddalidimerine; 11, toddayanis; and 12, zanthocadinanine A.

Caco-2 permeability also affects the GI permeability of the orally taken drugs. Its reference range is greater than 8 × 10^−6^ cm/s, and in the case of the pkCSM online tool, its normal value is >0.9. Isatithioetherin C, reserpine, toddayanis, and zanthocadinanine A had the Caco-2 value within this reference range. All the remaining metabolites, along with the standard, remdesivir, had a lower-than-reference range of Caco-2. Seven metabolites, namely, ergosine, isatithioetherin C, N,alpha-L-rhamnopyranosyl vincosamide, pelosine, reserpine, toddayanis, and zanthocadinanine, showed better Caco-2 permeability than the standard (Table 3).

P-glycoprotein acts as a housekeeping protein that plays a central role in drug absorption. P-glycoprotein caused less drug absorption. However, the inhibitors of P-glycoprotein have increased bioavailability as compared with non-inhibitor drugs (Finch and Pillans, 2014). Evodiamide C, toddalidimerine, and zanthocadinanine A did not act as substrates of P-glycoprotein (Table 3). The remaining nine tested metabolites, along with the standard, act as substrates for P-glycoprotein. All the tested metabolites, except isatithioetherin C, act as inhibitors of P-glycoprotein I and P-glycoprotein II. Isatithioetherin C did not act as an inhibitor of P-glycoproteins I and II. Remdesivir acts as an inhibitor of P-glycoprotein I (Table 3).

#### 3.9.2 Distribution parameter analysis

Parameters that affect drug distribution are the blood–brain barrier, central nervous system permeability, and steady-state volume of distribution (Han et al., 2019). The steady-state volume of distribution is directly related to the half-life of a drug. The shorter the VDss, the shorter the half-life of a drug, and a more frequent dose will be required (Holford and Yim, 2022). A drug that has a high volume of distribution will distribute more in the tissues as compared with the plasma. A low VDss value is below 0.15 log L/kg, and a high VDss value is above 0.45 log L/kg. Of the lead alkaloids sorted in this study, ergosine showed a high VDss of 1.139 log L/kg, and evodiamide C showed a low VDss of −1.481 log L/kg. The results indicated that ergosine, reserpine, toddayanis, and zanthocadinanine showed considerable VDss as compared with standard, in which VDss was 0.307 log L/kg, whereas the remaining eight metabolites showed low VDss (Table 3).

The molecules can cross the BBB if their logBB is greater than 0.3. If the logBB is less than −1, the molecule cannot cross the blood–brain barrier. None of the 12 selected metabolites, as well as the standard remdesivir, can cross the BBB. The central nervous system (CNS) permeability depends on the logPS value. The log PS >−2 to <−3 is the CNS permeability range (Murugesan et al., 2021). Evodiamide C, isatithioetherin C, reserpine, and N,alpha-L-rhamnopyranosyl vincosamide were found to be unable to cross the CNS. Remdesivir is also unable to cross the CNS (logPS = −4.675). The remaining eight alkaloids lie within the CNS permeability range (log PS > −2 to <−3; Table 3).

The pharmacodynamic and pharmacokinetic properties of a drug are related to the unbound fraction of the drug. Only this unbound drug fraction interacts with target proteins. The drug’s ability to diffuse inside the cell is directly related to the unbound drug fraction. The greater the unbound fraction of a drug, the greater its ability to diffuse (Watanabe et al., 2018). Remdesivir’s unbound fraction is 0.005 Fu. All 12 lead metabolites have a higher unbound fraction as compared with the standard. The unbound fraction ranges from 0.448 Fu (for toddalidimerine) to 0.019 Fu (for reserpine; Table 3).

#### 3.9.3 Metabolism parameter analysis

Cytochrome p450 enzymes play a key role in drug metabolism (Zanger et al., 2013). Five different cytochrome p450 enzymes (CYP2D6, CYP2C9, CYP1A2, CYP3A4, and CYP2C19) were considered for the metabolism analysis of leads. CYP3A4 is one of the most important members of the metabolic enzyme family that is involved in the metabolism of half of the drugs that are used these days (Guengerich, 2003). All the leads and standards did not act as substrates for CYP2D6. Except for N,alpha-L-rhamnopyranosyl vincosamide, all leads and standards acted as substrates for CYP3A4. They did not act as inhibitors of CYP1A2 and CYP2D6. Adouetine Y, evodiamide C, and homoaromoline inhibit CYP2C19, but the other eight metabolites do not act as inhibitors of CYP2C19. Except for adouetine Y and evodiamide C, all leads, along with the standard, did not inhibit CYP2C9. CYP3A4 is inhibited by adouetine Y, ergosine, evodiamide C, and reserpine (Table 3).

#### 3.9.4 Excretion parameter analysis

Total clearance of the drug plays a central role in choosing the dosing rate of the drug. The standard total clearance is 0.198 log mL/min/kg. The total clearance of the leads ranges from 0.766 (evodiamide C) to −0.565 (toddayanis). Hayatinine and pelosine acted as the renal OCT2 substrates. Except for these two metabolites, none of the other lead metabolites acted as substrates for renal OCT2. OCT2 is an important transporter that plays a central role in the excretion of endogenous compounds and drugs through urine (Burckhardt and Wolff, 2000). These metabolites cannot easily be excreted from the kidney because they are not acting as OCT2 substrates. As expected, none of them will be eliminated from the body through urination. These metabolites seem to be cleared out of the body through some other route (Table 3).

### 3.10 Toxicity parameter analysis

The toxicity of the compound is defined by certain parameters like hepatotoxicity, *Tetrahymena pyriformis* toxicity, AMES toxicity, maximum tolerance dose, hERG II inhibitor, oral rat chronic toxicity (LOAEL), hERG I inhibitor, and oral rat acute toxicity (LD50). All 12 lead metabolites did not cause skin sensitivity. Remdesivir did not show any AMES toxicity, hERG I inhibition, or skin sensitivity. Only three lead metabolites, namely, hayatinine, homoaromoline, and pelosine, cause AMES toxicity (Table 3). The AMES toxicity is related to the mutagenicity and carcinogenicity associated with the compound.

Adouetine Y, evodiamide C, and isatithioetherin C can induce hepatotoxicity. Remdesivir is also a hepatotoxicity-causing drug. Except for these three lead compounds, all other metabolites are safe and do not induce hepatotoxicity. The hER gene is directly linked with cardiotoxicity (Table 3). Thus, it is one of the important parameters for the toxicity evaluation of proposed drug candidates. Inhibition of hERg results in a disruption of cardiac rhythm that can be fatal (Tristani-Firouzi et al., 2001; Sanguinetti and Tristani-Firouzi, 2006).

All leads, along with the standard, act as hERG I inhibitors but do not act as hERG II inhibitors except isatithioetherin C. Acute (LD50) and chronic (LOAEL) toxicity analyses were conducted to ensure compound safety when administered. The standard exhibited an LD50 value of 2.043 mol/kg and an LOAEL value of 1.63 log mg/kgbw/day. The LD50 value ranges from 2.52 mol/kg (pelosine) to 3.29 mol/kg (zanthocadinanine A). Similarly, LOAEL ranges from −0.94 log mg/kgbw/day (toddalidimerine) to 0.90 log mg/kgbw/day (zanthocadinanine A; Table 3).

## 4 Conclusion

COVID-19 was one of the worst pandemics in the history of mankind. There is no proper treatment that could claim 100% protection from this deadly virus. This study exploited the antiviral potential of some selected medicinal plants and found that the alkaloids of *C*. *pareira* L. [Menispermaceae], *W*. *indica* L. [Malvaceae], *R*. *apiculata* Bl. [Rhizophoraceae], *E*. *rutaecarpa* (Juss.) Benth. [Rutaceae], *I. indigotica* Fort. [Brassicaceae], *M*. *oleifera* Lam. [Moringaceae], and *T*. *asiatica* (L.) Lam. [Rutaceae] showed good binding interactions with selected targets. The top 12 lead metabolites identified from these seven plants were adouetine Y, ergosine, evodiamide C, hayatinine, homoaromoline, isatithioetherin C, N-alpha-L-rhamnopyranosyl vincosamide, pelosine, reserpine, toddalidimerine, toddayanis, and zanthocadinanine. All these dual inhibitory alkaloids have more binding interactions than the selected standards.

Adouetine Y demonstrated the highest docking scores for both proteins; however, its positive total binding energy (MM-GBSA and MM-PBSA) suggests a nonspontaneous binding profile. Ergosine and evodiamide C were validated among the top three metabolites exhibiting favorable binding interactions in ligand–protein interaction analysis. MD simulations underscored the superior stability and binding affinity of adouetine Y, ergosine, and evodiamide C complexes with 6LU7 and 6LZG. In terms of bioactivity, adouetine Y, ergosine, and isatithioetherin C emerged as superior drug candidates. Most dual inhibitory alkaloids, such as adouetine Y and ergosine, showed good absorption and optimal bioavailability. All 12 lead metabolites exhibited a higher unbound fraction and therefore greater distribution compared with the standard. They demonstrated variable metabolic parameters and low excretion rates, and none caused skin sensitivity. Except for adouetine Y, evodiamide C, and isatithioetherin C, all other metabolites were non-hepatotoxic. Particularly, ergosine exhibited a stronger binding affinity similar to N3 and molnupiravir. These validated druggable alkaloids, ergosine and evodiamide C, investigated in this study have the potential to be evaluated for the discovery of a proper treatment against SARS-CoV-2.

Indeed, adouetine Y, our top metabolite, has been previously documented to have antiviral properties, which strengthens the findings of our computational analysis. Similarly, many other metabolites evaluated in our study have shown antiviral activities in various studies, which aligns with our predictions. The antiviral potential of the plants from which these alkaloids are derived, such as *C*. *pareira* L. [Menispermaceae] and *R. apiculata* Bl. [Rhizophoraceae], has also been established in previous research. This corroborates our findings and supports the hypothesis that these metabolites could be effective against viral infections, including SARS-CoV-2.

This study is limited by its *in silico* nature and lack of experimental validation, which limits the confirmation of the antiviral efficacy and safety of the identified metabolites. Future research should focus on conducting *in vitro* and *in vivo* studies to validate the antiviral potential of the identified alkaloids. Moreover, exploring their pharmacokinetics and potential combinatory effects with existing treatments could be valuable for developing effective COVID-19 therapies.

## Data Availability

The original contributions presented in the study are included in the article/Supplementary Material; further inquiries can be directed to the corresponding authors.
