# Supplementary material for: Identifying plant-derived antiviral alkaloids as dual inhibitors of SARS-CoV-2 main protease and spike glycoprotein through computational screening
Source: Front Pharmacol. 2024 Jul 17;15:1369659. doi: 10.3389/fphar.2024.1369659 (PMC11288853; doi:10.3389/fphar.2024.1369659)
Supplement: Supplementary file 1 [file Table1.docx]

**Table S1.** Alkaloids library isolated from selected antiviral plants.

| **Sr. No.** | **Compound** | **Plant Name** | **Docking Score Main Protease** | **Docking Score Spike Glycoprotein** | **Ref** |
| --- | --- | --- | --- | --- | --- |
|  | Pelosine  | *Cissampelos pareira* L. | 7.93 | -7.77 | [1] |
|  | Hayatine   | *C. pareira* L. | 7.82 | -7.43 | [1] |
|  | Hayatinine   | *C. pareira* L. | 8.11 | -7.91 | [2] |
|  | d-Isochondrodendrine   | *C. pareira* L. | 6.48 | -7.73 | [1] |
|  | Cissampareine   | *C. pareira* L. | 5.86 | -7.23 | [1] |
|  | Hayatidine   | *C. pareira* L. | 7.89 | -6.81 | [1] |
|  | Tetrandrine   | *C. pareira* L. | 7.92 | -7.30 | [3] |
|  | Cycleanine   | *C. pareira* L. | 7.98 | -8.24 | [4] |
|  | Insularine   | *C. pareira* L. | 7.51 | -7.69 | [1] |
|  | Sepeerine   | *C. pareira* L. | -7.78 | -7.30 | [1] |
|  | (+)-Obaberine   | *C. pareira* L. | -8.18 | -7.67 | [3] |
|  | (+)-Obamegine   | *C. pareira* L. | -6.77 | -8.00 | [3] |
|  | (+)-Homoaromoline   | *C. pareira* L. | 8.26 | -7.88 | [3] |
|  | (− )-nor-N′-chondrocurine   | *C. pareira* L. | -7.92 | -7.34 | [3] |
|  | 1-(4-(formyloxy)-3-  methoxybenzyl)-6,7-  dimethoxy-2,2-dimethyl-  1,2,3,4 tetrahydroisoquinolin-2-ium   | *C. pareira* L. | -7.42 | -5.93 | [4] |
|  | Magnocurarine   | *C. pareira* L. | -6.75 | -6.15 | [4] |
|  | (− )-Oblongine   | *C. pareira* L. | -6.21 | -6.03 | [3] |
|  | (+)-Coclaurine   | *C. pareira* L. | 6.24 | -5.94 | [3] |
|  | Pareirarine   | *C. pareira* L. | -6.74 | -6.77 | [2] |
|  | Dicentrine   | *C. pareira* L. | -6.53 | -6.29 | [1] |
|  | Dehydrodicentrine   | *C. pareira* L. | -6.53 | -6.56 | [1] |
|  | Magnoflorine   | *C. pareira* L. | -7.05 | -6.20 | [4], [2] |
|  | Pareirubrine A   | *C. pareira* L. | -6.73 | -6.53 | [4], [2] |
|  | Pareirubrine B   | *C. pareira* L. | -5.98 | -6.41 | [4], [2] |
|  | Isoimerubrine   | *C. pareira* L. | -6.76 | -6.96 | [1] |
|  | Grandirubrine   | *C. pareira* L. | -6.04 | -6.72 | [1] |
|  | Pareitropone   | *C. pareira* L. | -6.13 | -5.82 | [1] |
|  | Cissamine   | *C. pareira* L. | -6.17 | -5.75 | [1] |
|  | Berberine   | *C. pareira* L. | -6.45 | -6.15 | [1]  [5] |
|  | Norimeluteine   | *C. pareira* L. | -6.12 | -6.83 | [1] |
|  | Norruffscine   | *C. pareira* L. | -5.82 | -6.49 | [1] |
|  | Reserpine   | *C. pareira* L. | -8.84 | -9.17 | [1] |
|  | Cissampeline   | *C. pareira* L. | -7.01 | -6.66 | [3] |
|  | trans-N-feruloyltyramine   | *C. pareira* L. |  |  | [3] |
|  | Salutaridine   | *C. pareira* L. | -5.96 | -5.92 | [2] |
|  | Laudanosine   | *C. pareira* L. | -6.55 | -6.94 | [1] |
|  | Nuciferine   | *C. pareira* L. | -6.25 | -6.09 | [1] |
|  | Corytuberine   | *C. pareira* L. | -6.87 | -6.57 | [1] |
|  | Bulbocarpine   | *C. pareira* L. | -6.23 | -5.95 | [1] |
|  | N-methyl-magnoflorin   | *C. pareira* L. | -6.82 | -6.25 | [1] |
|  | 8- methoxynorchelerythrine   | *Toddalia asiatica* (L.) Lam | -6.89 | -6.09 | [6] |
|  | 11-demethylrhoifoline  B   | *T. asiatica* (L.) Lam | -6.53 | -6.57 | [6] |
|  | 8-methoxynitidine   | *T. asiatica* (L.) Lam | -6.37 | -6.45 | [6] |
|  | 8-acetylnorchelerythrine   | *T. asiatica* (L.) Lam | -7.30 | -6.15 | [6] |
|  | 8,9,10,12-tetramethoxynorchelerythrine   | *T. asiatica* (L.) Lam | -7.03 | -7.59 | [6] |
|  | Isointegriamide   | *T. asiatica* (L.) Lam | -7.18 | -6.51 | [6] |
|  | 1-demethyl dicentrinone   | *T. asiatica* (L.) Lam | -6.33 | -6.35 | [6] |
|  | 11-hydroxy-10-methoxy-(2,3)-  Methylenedioxytetrahydroproto- berberine   | *T. asiatica* (L.) Lam | -6.71 | -6.46 | [6] |
|  | rhoifoline B   | *T. asiatica* (L.) Lam | -6.69 | -6.68 | [6] |
|  | Pancorine   | *T. asiatica* (L.) Lam | -6.28 | -5.87 | [6]  [7] |
|  | 8-methoxychelerythrine   | *T. asiatica* (L.) Lam | -6.43 | -6.22 | [6] |
|  | Arnottianamide   | *T. asiatica* (L.) Lam | -7.23 | -6.68 | [6] |
|  | oxynitidine   | *T. asiatica* (L.) Lam | -6.2504 | -6.6301 | [6]  [8] |
|  | Oxysanguinarine   | *T. asiatica* (L.) Lam | -6.0893 | -6.2904 | [6] |
|  | Dicentrinone   | *T. asiatica* (L.) Lam | -6.3661 | -6.2469 | [6] |
|  | (2,3,10,11)-dimethylenedioxytetrahydroprotoberberine   | *T. asiatica* (L.) Lam | -7.0805 | -5.9287 | [6] |
|  | Skimmianine   | *T. asiatica* (L.) Lam | -6.1265 | -5.6944 | [6] |
|  | 5-methoxydictamnine   | *T. asiatica* (L.) Lam | -5.5346 | -4.9129 | [6] |
|  | Robustine   | *T. asiatica* (L.) Lam | -5.4826 | -5.1011 | [9] |
|  | 8-Acetonyldihydrochelerythrine   | *T. asiatica* (L.) Lam | -6.4492 | -6.7318 | [9]  [8] |
|  | Toddalidimerine   | *T. asiatica* (L.) Lam | -8.8428 | -10.0282 | [9] |
|  | 8-Methoxydihydrochelerythrine   | *T. asiatica* (L.) Lam | -6.7926 | -6.3412 | [9] |
|  | 2,3-Dimethoxy-6-(5-(N-methylformamido)naphtho[2,3-d][1,3]dioxol-6-yl)phenyl  Acetate   | *T. asiatica* (L.) Lam | -7.5457 | -7.2308 | [9] |
|  | 8-Acetoxydihydrochelerythrine   | *T. asiatica* (L.) Lam | -6.4048 | -6.9845 | [9] |
|  | Chelerythrine   | *T. asiatica* (L.) Lam | -6.6338 | -6.2378 | [9]  [10] |
|  | 4-Methoxy-1-methylquinolin-2-one   | *T. asiatica* (L.) Lam | -5.2818 | -4.4627 | [9] |
|  | Oxychelerythrin   | *T. asiatica* (L.) Lam | -6.6304 | -6.3878 | [9] |
|  | Integriquinolone   | *T. asiatica* (L.) Lam | -5.3875 | -4.5623 | [9] |
|  | N-methylflindersine   | *T. asiatica* (L.) Lam | -6.0761 | -5.2293 | [9] |
|  | Oxyavicine   | *T. asiatica* (L.) Lam | -6.8552 | -5.8818 | [9]  [8] |
|  | Avicine   | *T. asiatica* (L.) Lam | -5.9277 | -5.8514 | [9] |
|  | Toddaquinoline   | *T. asiatica* (L.) Lam | -5.4915 | -5.0306 | [9] |
|  | Norchelerythrine   | *T. asiatica* (L.) Lam | -6.2941 | -5.9152 | [9] |
|  | Toddayanis   | *T. asiatica* (L.) Lam | -9.5061 | -8.4019 | [9] |
|  | Haplopine   | *T. asiatica* (L.) Lam | -5.8305 | -5.6117 | [9]  [8] |
|  | Isocoreximine   | *T. asiatica* (L.) Lam | -6.7652 | -6.7342 | [9] |
|  | Oxyterihannine   | *T. asiatica* (L.) Lam | -6.3951 | -6.3078 | [9] |
|  | Chelerythrine chlorid   | *T. asiatica* (L.) Lam |  |  | [9]  [11] |
|  | Nitidine chloride   | *T. asiatica* (L.) Lam |  |  | [9]  [11] |
|  | Flindersine   | *T. asiatica* (L.) Lam | -5.5458 | -4.7528 | [12] |
|  | 3-(2,3-Dihydroxy-3-methylbutyl)-4,7-dimethoxy-1-methyl-1H-quinolin-2-one   | *T. asiatica* (L.) Lam | -7.0486 | -6.3888 | [9]  [11] |
|  | N-methyl-4-hydroxy-7-methoxy-3-(2,3-epoxy-3-methylbutyl)-1H-quinolin-2-one   | *T. asiatica* (L.) Lam | -6.2326 | -5.8557 | [9]  [11] |
|  | Demethylnitidine   | *T. asiatica* (L.) Lam | -6.5168 | -5.8571 | [13] |
|  | Dihydroavicine   | *T. asiatica* (L.) Lam | -6.1318 | -5.8022 |  |
|  | Dihydrochelerythrine   | *T. asiatica* (L.) Lam | -6.6766 | -6.3825 | [8] |
|  | Dihydronitidine   | *T. asiatica* (L.) Lam | -6.9024 | -6.4150 | [14] |
|  | Zanthocadinanine A   | *T. asiatica* (L.) Lam | -8.1052 | -7.9626 | [15] |
|  | Protopine   | *T. asiatica* (L.) Lam | -6.5562 | -6.2168 | (Lin et al., 2014  [10] |
|  | 4-Hydroxy-N-methylproline   | *T. asiatica* (L.) Lam |  |  | [16] |
|  | Dictamnine   | *T. asiatica* (L.) Lam | -5.2188 | -4.7435 | [16]  [8] |
|  | 8-hydroxy-dihydrochelerythrine   | *T. asiatica* (L.) Lam | -6.9494 | -6.3210 | [17] |
|  | Nitidine   | *T. asiatica* (L.) Lam | -6.9706 | -6.4709 | [17] |
|  | Pioglitazone   | *T. asiatica* (L.) Lam | -7.2850 | -6.3913 | [18] |
|  | N-cis-feruloyltyramide   | *T. asiatica* (L.) Lam | -6.8482 | -6.0508 | [19] |
|  | 8S-10-O-demethylbocconoline   | *T. asiatica* (L.) Lam | -6.4289 | -6.4318 | [20] |
|  | 8-Acetonyldihydroavicine   | *T. asiatica* (L.) Lam | -7.7267 | -6.9175 | [21] |
|  | 8-Acetonyldihydronitidine   | *T. asiatica* (L.) Lam | -7.7677 | -7.3391 | [21] |
|  | Decarine   | *T. asiatica* (L.) Lam | -6.1330 | -5.8067 | [21]  [8] |
|  | desformyl-isoarnottianamide   | *T. asiatica* (L.) Lam | -7.2760 | -6.3664 | [8] |
|  | Noravicine   | *T. asiatica* (L.) Lam | -6.1384 | -5.6491 | [8] |
|  | zanthoxyline dimethoxy derivative | *T. asiatica* (L.) Lam | -6.3313 | -6.6192 | [8] |
|  | N-nornitidine | *T. asiatica* (L.) Lam | -6.6469 | -6.1830 | [8] |
|  | γ-fagarine | *T. asiatica* (L.) Lam | -5.5911 | -4.8637 | [8] |
|  | Oxynorchelerythrin | *T. asiatica* (L.) Lam |  |  | [20] |
|  | Picrinine | *Alstonia scholaris* (L.) R.Br. |  |  | [22] |
|  | Scholaricine | *A. scholaris* (L.) R.Br. | -5.8960 | -6.4632 | [22] |
|  | Vallesamine | *A. scholaris* (L.) R.Br. | -6.3346 | -5.8979 | [22] |
|  | 19-epischolaricine | *A. scholaris* (L.) R.Br. | -6.2779 | -6.6413 | [22] |
|  | Alstoscholarisines A | *A. scholaris* (L.) R.Br. |  |  | [23] |
|  | Alstoscholarisines B | *A. scholaris* (L.) R.Br. |  |  | [23] |
|  | Alstoscholarisines C | *A. scholaris* (L.) R.Br. | -6.3166 | -5.7754 | [23] |
|  | Alstoscholarisines D | *A. scholaris* (L.) R.Br. | -5.5435 |  | [23] |
|  | Alstoscholarisines E | *A. scholaris* (L.) R.Br. | -6.1636 | -5.4366 | [23] |
|  | Alstoscholarisine F | *A. scholaris* (L.) R.Br. | -5.1167 | -5.4698 | [24] |
|  | Alstoscholarisines G | *A. scholaris* (L.) R.Br. | -6.9333 | -6.2270 | [24] |
|  | Alstoscholarisines H | *A. scholaris* (L.) R.Br. | -5.9552 | -5.8908 | [25] |
|  | Alstoscholrisines I | *A. scholaris* (L.) R.Br. |  |  | [25] |
|  | Alstoscholarisines J | *A. scholaris* (L.) R.Br. |  |  | [25] |
|  | Nicotine | *A. scholaris* (L.) R.Br. | -5.3456 | -4.7981 | [26] |
|  | Strictamine | *A. scholaris* (L.) R.Br. | -5.1792 | -5.1778 | [26] |
|  | Voacristine | *A. scholaris* (L.) R.Br. | -7.1187 | -6.3003 | [26] |
|  | (E)-Akuammidine | *A. scholaris* (L.) R.Br. | -6.4927 |  | [26] |
|  | (Z)-Akuammidine | *A. scholaris* (L.) R.Br. |  |  | [26] |
|  | nareline ethyl ether | *A. scholaris* (L.) R.Br. |  |  | [27] |
|  | 5-epi-nareline ethyl ether | *A. scholaris* (L.) R.Br. | -6.3765 | -5.2507 | [27] |
|  | scholarine-N (4)-  oxide | *A. scholaris* (L.) R.Br. |  | -5.2144 | [27] |
|  | nareline methyl ether | *A. scholaris* (L.) R.Br. | -5.1809 |  | [27] |
|  | scholarisine I | *A. scholaris* (L.) R.Br. | -6.0264 | -5.1927 | [28] |
|  | (±)-scholarisine II | *A. scholaris* (L.) R.Br. | -5.6838 | -5.2015 | [28] |
|  | (19,20) Z-alstoscholarine | *A. scholaris* (L.) R.Br. | -6.1029 | -6.5181 | [29] |
|  | (19,20) E-alstoscholarine | *A. scholaris* (L.) R.Br. | -6.0931 | -6.2524 | [29] |
|  | 19-epi-scholarisine | *A. scholaris* (L.) R.Br. | -5.2887 | -5.9060 | [29] |
|  | N4-methylscholaricine | *A. scholaris* (L.) R.Br. | -5.8395 | -6.3411 | [30] |
|  | 12-hydroxy-echitamidine N-oxide | *A. scholaris* (L.) R.Br. | -5.3138 |  | [30] |
|  | Echitamidine | *A. scholaris* (L.) R.Br. | -5.2427 | -6.0368 | [30] |
|  | N-demethylalstogustine | *A. scholaris* (L.) R.Br. | -5.5768 | -6.0536 | [30] |
|  | Lagunamine | *A. scholaris* (L.) R.Br. |  |  | [30] |
|  | Vallesamine N-oxide | *A. scholaris* (L.) R.Br. | -5.9023 |  | [30] |
|  | Losbanine | *A. scholaris* (L.) R.Br. | -5.8967 | -6.2916 | [30] |
|  | N4-methyl angusilobine B | *A. scholaris* (L.) R.Br. | -6.6235 | -5.7840 | [30] |
|  | Akuammidine | *A. scholaris* (L.) R.Br. | -6.9779 |  | [30] |
|  | N4-demethylechitamine | *A. scholaris* (L.) R.Br. |  |  | [30] |
|  | Burnamine | *A. scholaris* (L.) R.Br. |  |  | [30] |
|  | 5-methoxystrictamine | *A. scholaris* (L.) R.Br. | -5.2940 | -3.9192 | [30] |
|  | Picralinal | *A. scholaris* (L.) R.Br. |  | -5.1436 | [30] |
|  | methyl (16R,19E)-1,2-dihydro-16-(hydroxymethyl)-5-oxoakuammilan-17-  oate | *A. scholaris* (L.) R.Br. |  |  | [31] |
|  | methyl (2 β ,16R,19E)-4,5-didehydro-1,2-dihydro-2-hydroxy-16-(hydroxymethyl)akuammilan-4-ium-17-oate chloride | *A. scholaris* (L.) R.Br. |  |  | [31] |
|  | Rhazimanine | *A. scholaris* (L.) R.Br. | -6.7342 | -7.3642 | [31] |
|  | Scholarisines G | *A. scholaris* (L.) R.Br. | -5.7279 | -5.2066 | [32] |
|  | Leuconoxine | *A. scholaris* (L.) R.Br. | -6.1665 | -5.4613 | [32] |
|  | 5-methoxylstrictamine | *A. scholaris* (L.) R.Br. | -5.5928 | -5.6866 | [33]  [32] |
|  | 19-epi-ajmalicine | *A. scholaris* (L.) R.Br. | -7.2067 | -6.4007 | [32] |
|  | Echitamine | *A. scholaris* (L.) R.Br. | -4.6459 | -3.6645 | [32] |
|  | Alstorisine A | *A. scholaris* (L.) R.Br. | -5.4088 |  | [34] |
|  | Vincadifformine | *A. scholaris* (L.) R.Br. | -6.9270 | -6.3508 | [34] |
|  | Vallesiachotamine | *A. scholaris* (L.) R.Br. | -7.1486 | -6.5209 | [34] |
|  | Isovallesiachotamine | *A. scholaris* (L.) R.Br. | -7.2626 | -6.6602 | [34] |
|  | tubotaiwine N-oxide | *A. scholaris* (L.) R.Br. |  | -3.1874 | [34] |
|  | 6,7-secoangustilobine B | *A. scholaris* (L.) R.Br. | -7.3542 | -6.4853 | [35] |
|  | angustilobine B | *A. scholaris* (L.) R.Br. | -6.3016 | -5.8704 | [35] |
|  | Manilamine | *A. scholaris* (L.) R.Br. | -6.5902 | -6.2280 | [35] |
|  | Tubotaiwine | *A. scholaris* (L.) R.Br. |  |  | [35] |
|  | Harmaline | *Peganum harmala* L. | -5.8079 | -4.7366 | [36] |
|  | Harmine | *P. harmala* L. | -5.4732 | -5.0551 | [36] |
|  | Harmalol | *P. harmala* L. | -5.1304 | -5.0807 | [36] |
|  | Harman | *P. harmala* L. | -4.9801 | -4.3606 | [36] |
|  | Tetrahydroharmine | *P. harmala* L. | -5.8817 | -4.9263 | [36] |
|  | Harmol | *P. harmala* L. | -5.1400 | -4.6524 | [36] |
|  | Deoxypeganine | *P. harmala* L. | -5.0957 | -4.0136 | [36] |
|  | Deoxyvasicinone | *P. harmala* L. | -5.1364 | -4.7831 | [36] |
|  | Vasicine | *P. harmala* L. | -5.3851 | -5.2778 | [36] |
|  | Pegamine | *P. harmala* L. | -5.4638 | -4.9167 | [36] |
|  | Peganine | *P. harmala* L. | -5.3808 | -5.2702 | [37] |
|  | Harmalicin | *P. harmala* L. | -5.5439 | -5.0331 | [38] |
|  | 3,4-dihydroharmane | *P. harmala* L. | -5.3000 | -4.4750 | [38] |
|  | 7-hydroxy-3,4-dihydronorharmin-1-one | *P. harmala* L. | -5.3364 | -4.5239 | [38] |
|  | Harmalanine | *P. harmala* L. | -6.1605 | -5.7605 | [38] |
|  | Harmalacidine | *P. harmala* L. | -5.5527 | -5.0602 | [38] |
|  | Peganol | *P. harmala* L. | -5.2106 | -4.7616 | [38] |
|  | Peganidine | *P. harmala* L. | -5.5416 | -5.3689 | [38] |
|  | Vazicinone | *P. harmala* L. | -5.3410 | -4.8412 | [38] |
|  | Deoxyvazicinone | *P. harmala* L. | -5.1242 | -4.3603 | [38] |
|  | 4-Butyl pyridine, 1-oxide | *Rhizophora apiculata* Blume | -5.4672 | -4.6196 | [39] |
|  | Clivorine | *R. apiculata* Blume | -5.8806 | -6.3303 | [39] |
|  | Muscimol | *R. apiculata* Blume | -4.4113 | -3.5387 | [40] |
|  | Glycosin | *R. apiculata* Blume | -6.0121 | -5.6349 | [40] |
|  | Ergotamine | *R. apiculata* Blume | -6.4743 | -5.9226 | [40] |
|  | Ergobasine | *R. apiculata* Blume | -4.3865 | -3.7445 | [40] |
|  | Ergosine | *R. apiculata* Blume | -8.1190 | -7.7862 | [40] |
|  | Mescaline | *R. apiculata* Blume | -5.8736 | -5.1315 | [40] |
|  | Pilosine | *R. apiculata* Blume | -6.9646 | -6.0108 | [40] |
|  | Peganumine D | *P. harmala* L. | -6.1642 | -5.9548 | [41] |
|  | S-Peganumine E | *P. harmala* L. | -5.9209 | -5.1458 | [41] |
|  | R-Peganumine E | *P. harmala* L. | -6.3939 | -5.3697 | [41] |
|  | Peganumine F | *P. harmala* L. | -6.7210 | -7.0305 | [41] |
|  | Peganumine G | *P. harmala* L. | -6.5447 | -6.9486 | [41] |
|  | (R)-vasicinone-1-O-𝛽-D-glucopyranoside | *P. harmala* L. | -6.3774 | -7.1028 | [42] |
|  | (S)-vasicinone | *P. harmala* L. | -5.3033 | -4.6014 | [42] |
|  | (S)-vasicinone-1-O-𝛽-D-glucopyranoside | *P. harmala* L. | -6.9082 | -6.7499 | [42] |
|  | Serpentine | *Rhizophora mucronata* Poir. | -7.0782 | -6.0744 | [43] |
|  | Catharanthine | *R. mucronate* Poir. | -6.4894 | -6.2739 | [43] |
|  | Vindoline | *R. mucronate* Poir. | -6.8516 | -6.7150 | [43] |
|  | Ajmalicine | *R. mucronate* Poir. | -7.2102 | -6.4526 | [43] |
|  | isatithioetherins A | *Isatis indigotica* Fort. | -8.1191 | -7.4508 | [44] |
|  | isatithioetherins B | *I. indigotica* Fort. | -8.3260 | -7.7180 | [44] |
|  | isatithioetherins C | *I. indigotica* Fort. | -8.3274 | -7.8499 | [44] |
|  | isatithioetherins D | *I. indigotica* Fort. | -7.8640 | -7.9838 | [44] |
|  | isatithioetherins E | *I. indigotica* Fort. | -7.4119 | -7.3885 | [44] |
|  | Indigotine | *I. indigotica* Fort. | -5.6033 | -5.3446 | [45] |
|  | Indirubin | *I. indigotica* Fort. | -5.8755 | -5.5678 | [45] |
|  | Isaindigotone | *I. indigotica* Fort. | -7.0462 | -6.1096 | [45] |
|  | Tryptanthrin | *I. indigotica* Fort. | -5.5267 | -5.1768 | [45] |
|  | 2,5-dihydroxy-indole | *I. indigotica* Fort. | -4.6386 | -4.3245 | [45] |
|  | 2,3-dihydro-4-hydroxy-2-oxo-indole-3-acetonitrile | *I. indigotica* Fort. | -4.3933 | -4.4544 | [45] |
|  | Hydroxyindirubin | *I. indigotica* Fort. | -5.4634 | -5.5714 | [45] |
|  | Isatin | *I. indigotica* Fort. | -4.4077 | -3.9140 | [45] |
|  | 2,4(1H,3H)-quinazolinedion | *I. indigotica* Fort. | -4.8188 | -4.0647 | [45] |
|  | 5-hydroxy-2-indolinone | *I. indigotica* Fort. | -4.1499 | -4.3079 | [45] |
|  | 10H-indole[3,2-b]quinolone | *I. indigotica* Fort. | -5.2564 | -4.8520 | [45] |
|  | Isatan A | *I. indigotica* Fort. | -6.6374 | -6.4485 | [45] |
|  | 3-formyl-indole | *I. indigotica* Fort. | -4.7450 | -4.0014 | [45] |
|  | Deoxyvascinone | *I. indigotica* Fort. | 5.1236 | -4..3399 | [45] |
|  | 4(3H)-quinazolinone | *I. indigotica* Fort. | -4.6429 | -3.9482 | [45] |
|  | 3-(2'-hydroxyphenyl)-4(3H)-quinazolinone | *I. indigotica* Fort. | -5.7123 | -5.1355 | [45] |
|  | 3-dihydro-1H-pyrrolo[2,1-c][1,4]benzodiazepine-5,11(10H,11aH)-dione | *I. indigotica* Fort. | -5.4810 | -5.2655 | [45] |
|  | (E)-3-(3',5'-dimethoxy-4'-hydroxybenzylidene)-2-indolinone | *I. indigotica* Fort. | -6.7392 | -5.7328 | [45] |
|  | Isaindigotidione | *I. indigotica* Fort. | -7.6740 | -7.3546 | [46]  [47] |
|  | indole-3-acetonitrile-2-S-β-D-glucopyranoside | *I. indigotica* Fort. | -6.9836 | -6.1909 | [48] |
|  | indole-3-  acetonitrile-4-methoxy-2-S-β-D-glucopyranoside | *I. indigotica* Fort. | -6.0167 | -6.3785 | [48] |
|  | N-methoxy-indole-3-acetonitrile-  2-S-β-D-glucopyranoside | *I. indigotica* Fort. | -7.2984 | -7.2172 | [48] |
|  | indole-3-acetonitrile-6-O-β-D-glucopyranoside | *I. indigotica* Fort. | -7.1558 | -6.0916 | [48] |
|  | indole3-acetonitrile | *I. indigotica* Fort. | -5.2751 | -4.3779 | [48] |
|  | Arvelexin | *I. indigotica* Fort. | -5.5712 | -4.7802 | [48] |
|  | 1-methoxy-indole-3-  Acetonitrile | *I. indigotica* Fort. | -6.0779 | -4.8644 | [48] |
|  | 3-indoleformic acid | *I. indigotica* Fort. | -5.1000 | -4.2721 | [48] |
|  | 3-indoleformic  acid methyl ester | *I. indigotica* Fort. | -5.2520 | -4.2266 | [48] |
|  | 1-methoxy-3-indoleformic acid | *I. indigotica* Fort. | -5.8713 | -4.7909 | [48] |
|  | 3-indoleacetic acid | *I. indigotica* Fort. | -5.3477 | -4.5669 | [48] |
|  | 4-methoxy-3-indoleacetic  Acid | *I. indigotica* Fort. | -5.4920 | -5.0923 | [48] |
|  | 1-methoxy-3-indoleacetic acid | *I. indigotica* Fort. | -5.7488 | -5.4135 | [48] |
|  | 1-methoxy-3-indolecarbaldehyde | *I. indigotica* Fort. | -5.3852 | -4.3819 | [48] |
|  | waltheriones E | *Waltheria indica* L. | -7.3356 | -6.8924 | [49] |
|  | waltheriones F | *W. indica* L. | -6.8936 | -6.9283 | [49] |
|  | waltheriones G | *W. indica* L. |  |  | [49] |
|  | waltheriones H | *W. indica* L. | -7.9415 | -7.6400 | [49] |
|  | waltherione I | *W. indica* L. | -7.6941 | -6.8692 | [49] |
|  | waltheriones J | *W. indica* L. | -7.3625 | -7.0563 | [49] |
|  | waltheriones K | *W. indica* L. | -7.6396 | -7.0420 | [49] |
|  | waltheriones L | *W. indica* L. | -6.6709 | -7.0468 | [49] |
|  | 8-  Deoxoantidesmone | *W. indica* L. | -7.3096 | -6.2924 | [49] |
|  | Antidesmone | *W. indica* L. | -7.0394 | -6.6068 | [49] |
|  | Adouetine X | *W. indica* L. | -7.5495 | -7.5534 | [50] |
|  | Adouetine Y | *W. indica* L. | -8.4095 | -8.6643 | [50] |
|  | Adouetine Z | *W. indica* L. | -8.7289 | -8.7666 | [50] |
|  | Waltherione M | *W. indica* L. | -6.9946 | -6.7742 | [49] |
|  | Waltheriones N | *W. indica* L. | -7.6951 | -6.6842 | [49] |
|  | Waltheriones O | *W. indica* L. | -7.6584 | -6.4197 | [49] |
|  | Waltheriones P | *W. indica* L. | -7.3935 | -6.9146 | [49] |
|  | Waltheriones Q | *W. indica* L. | -7.1467 | -7.3445 | [49] |
|  | 5(R)-vanessine | *W. indica* L. | -7.7501 | -6.3002 | [49] |
|  | 8-demethoxywaltherione F | *W. indica* L. | -6.9608 | -6.4218 | [51] |
|  | Waltherione R | *W. indica* L. | -7.2613 | -7.3729 | [51] |
|  | Waltherione S | *W. indica* L. | -7.6276 | -6.4056 | [51] |
|  | Waltherione T | *W. indica* L. | -7.2788 | -7.3819 | [51] |
|  | Waltherione U | *W. indica* L. | -7.7407 | -7.2658 | [51] |
|  | Waltherione V | *W. indica* L. |  |  | [51] |
|  | 1-methoxywaltherione O | *W. indica* L. | -7.8127 | -7.3878 | [51] |
|  | (S)-15-hydroxywaltherione G | *W. indica* L. | -7.2233 | -7.1955 | [51] |
|  | (8R)-8-hydroxywaltherione M | *W. indica* L. | -7.4942 | -7.5062 | [51] |
|  | (9S,13S)-2-  hydroxymethylwaltherione C | *W. indica* L. | -7.4914 | -6.5967 | [51] |
|  | (9S,10S,13S)-10-hydroxywaltherione C | *W. indica* L. | -7.2267 | -6.7975 | [51]v |
|  | (S)-13-methoxywaltherione V | *W. indica* L. | -7.5771 | -6.2088 | [51]v |
|  | Melovinone | *W. indica* L. | -7.5115 | -8.1308 | [51] |
|  | 5′-methoxywaltherione A | *W. indica* L. | -7.0386 | -6.4312 | [51] |
|  | (+)-argenaxine | *Argemone Mexicana* L. | -6.7792 | -6.6283 | [7] |
|  | (+)-higenamine | *A. Mexicana* L. | -5.9188 | -5.9921 | [7] |
|  | (+)-reticuline | *A. Mexicana* L. | -6.7787 | -6.9375 | [7] |
|  | isocorydine | *A. Mexicana* L. | -6.8746 | -6.7522 | [5] |
|  | Dehydrocheilanthifoline | *A. Mexicana* L. | -6.7400 | -6.0076 | [10] |
|  | Dehydrocorydalmine | *A. Mexicana* L. | -6.5537 | -6.3199 | [52] |
|  | Jatrorrhizine | *A. Mexicana* L. | -6.7741 | -6.5083 | [52] |
|  | Columbamine | *A. Mexicana* L. | -6.6514 | -6.3575 | [52] |
|  | Coptisine | *A. Mexicana* L. | -5.8477 | -5.7021 | [10] |
|  | Allocryptopine | *A. Mexicana* L. | -7.0531 |  | [10] |
|  | cryptopine | *A. Mexicana* L. | -6.0807 | -6.7682 | [5] |
|  | Muramine | *A. Mexicana* L. | -6.5057 | -7.3949 | [5] |
|  | argemexicaine A | *A. Mexicana* L. | -7.0702 | -6.9941 | [10] |
|  | argemexicaine B | *A. Mexicana* L. | -6.3742 | -6.4583 | [10] |
|  | Protomexicine | *A. Mexicana* L. | -6.9995 | -6.8281 | [53] |
|  | 13-oxoprotopine | *A. Mexicana* L. | -5.8252 | -5.5882 | [53] |
|  | (-)-scoulerine | *A. Mexicana* L. | -6.9282 | -6.6155 | [5] |
|  | (-)-stylopine | *A. Mexicana* L. | -6.8746 | -5.8323 | [5] |
|  | nor-sanguinarine | *A. Mexicana* L. | -6.1347 | -5.7273 | [5] |
|  | Sanguinarine | *A. Mexicana* L. | -6.2817 | -5.5528 | [10] |
|  | Oxyhydrastinine | *A. Mexicana* L. | -5.6896 | -4.5009 | [5] |
|  | Thalifoline | *A. Mexicana* L. | -5.2646 | -4.6538 | [5] |
|  | Argemexirine | *A. Mexicana* L. | -5.8316 | -5.4858 | [54] |
|  | higenamine | *A. Mexicana* L. | -5.7646 | -5.9737 | [7] |
|  | (±)-tetrahydrocoptisine | *A. Mexicana* L. | -6.8103 | -5.8251 | [54] |
|  | (-)-tetrahydroberberine | *A. Mexicana* L. | -6.6888 | -6.4646 | [10] |
|  | Dihydrocoptisine | *A. Mexicana* L. | -6.8171 | -6.2280 | [54] |
|  | Oxyberberine | *A. Mexicana* L. | -6.7637 | -6.3525 | [52] |
|  | N-demethyloxysanguinarine | *A. Mexicana* L. | -6.1085 | -5.8821 | [7] |
|  | (±)6-acetonyl  Dihydrochelerythrine | *A. Mexicana* L. | -7.6309 | -7.6102 | [10] |
|  | Dihydrosanguiranine | *A. Mexicana* L. | -6.5117 | -5.8829 | [7] |
|  | Angoline | *A. Mexicana* L. | -6.9413 | -6.4612 | [10], [7] |
|  | 8-acetonyl  Dihydrosanguiranine | *A. Mexicana* L. | -7.2065 | -6.3784 | [5] |
|  | 8-methoxy  Dihydrosanguiranine | *A. Mexicana* L. | -7.0568 | -6.4748 | [53] |
|  | dihydropalmatine hydroxide | *A. Mexicana* L. |  |  | [5] |
|  | (-)-argemonine | *A. Mexicana* L. | -6.1377 | -6.0325 | [5] |
|  | 1-methyl-2-nonyl-4(1H)-  Quinolone | *Evodia rutaecarpa* (Juss.) Benth. | -7.2187 | -6.1810 | [55] |
|  | 1-methyl-2-dodecyl-4(1H)-quinolone | *E. rutaecarpa* (Juss.) Benth. | -7.4846 | -6.9990 | [55] |
|  | 2-tridecyl-  4(1H)-quinolone | *E. rutaecarpa* (Juss.) Benth. | -7.2607 | -6.8333 | [55] |
|  | Dihydroevocarpine | *E. rutaecarpa* (Juss.) Benth. | -7.3413 | -7.4005 | [55] |
|  | 1-methyl-2-  pentadecyl-4(1H)-quinolone | *E. rutaecarpa* (Juss.) Benth. | -7.5877 | -6.7425 | [55] |
|  | 2-Undecanone-10’-4(1H)-quinolone | *E. rutaecarpa* (Juss.) Benth. | -7.3383 | -6.5213 | [56] |
|  | 1-Methyl-2-undecanone-10’-4(1H)-quinolone | *E. rutaecarpa* (Juss.) Benth. | -7.0333 | -6.5734 | (X. W. Yang et al., 2006 |
|  | 2-Undecyl-4(1H)-quinolone | *E. rutaecarpa* (Juss.) Benth. | -7.3345 | -6.3953 | (X. W. Yang et al., 2006 |
|  | 1-Methyl-2-undecyl-4(1H)-quinoline | *E. rutaecarpa* (Juss.) Benth. | -6.9337 | -6.6870 | (X. W. Yang et al., 2006 |
|  | Rutaecarpine | *E. rutaecarpa* (Juss.) Benth. | -5.3881 |  | (X. W. Yang et al., 2006 |
|  | evodiamide A | *E. rutaecarpa* (Juss.) Benth. | -7.5761 | -6.8309 | [57] |
|  | evodiamide B | *E. rutaecarpa* (Juss.) Benth. | -7.0270 | -6.2141 | [57] |
|  | evodiamide C | *E. rutaecarpa* (Juss.) Benth. | -9.1521 | -9.0502 | [57] |
|  | wuchuyuamide I | *E. rutaecarpa* (Juss.) Benth. | -7.1090 | -6.5842 | [57] |
|  | wuchuyuamide III | *E. rutaecarpa* (Juss.) Benth. | -6.2595 | -6.4109 | [57] |
|  | wuzhuyurutine B | *E. rutaecarpa* (Juss.) Benth. | -6.3668 | -5.6427 | [57] |
|  | Evodiamine | *E. rutaecarpa* (Juss.) Benth. | -6.4379 | -5.9932 | [57] |
|  | Rhetsinine | *E. rutaecarpa* (Juss.) Benth. | -6.4895 | -6.2935 | [57] |
|  | Evodiaxinine | *E. rutaecarpa* (Juss.) Benth. | -6.5624 | -5.9622 | [57] |
|  | Dehydroevodiamine | *E. rutaecarpa* (Juss.) Benth. | -5.8316 | -5.8112 | [57] |
|  | -2-undecyl-quinolone | *E. rutaecarpa* (Juss.) Benth. | 7.3133 | -6.9775 | [58] |
|  | methyl-2-[(4Z,7Z)-4,7-tridecadieny]-4(1H)-quinolone | *E. rutaecarpa* (Juss.) Benth. | -7.8331 | 6.9996 | [58] |
|  | Evocarpine | *E. rutaecarpa* (Juss.) Benth. | -7.1774 | -7.2307 | [58] |
|  | 1-methyl-2-[(6Z,9Z)-6,9-pentadecadienyl]-4(1H)-quinolone | *E. rutaecarpa* (Juss.) Benth. | -7.3249 | -7.4771 | [58] |
|  | wuchuyuamide Ⅳ | *E. rutaecarpa* (Juss.) Benth. | -6.6376 | -7.0266 | [58] |
|  | Confusamelin | *Dictamnus dasycarpus* Turcz. | -5.3659 | -5.2043 | [59] |
|  | O-Ethylnordictamnine | *D. dasycarpus* Turcz. | -5.5011 | -5.0218 | [59] |
|  | O-Ethylnor-γ-fagarine | *D. dasycarpus* Turcz. | -5.8291 | -5.3560 | [59] |
|  | O-Ethylnorskimmianine | *D. dasycarpus* Turcz. | -6.3687 | -5.5959 | [59] |
|  | 8-Hydroxy-9-methyl-furo[2,3-b]quinolin-4(9H)-on | *D. dasycarpus* Turcz. | -5.2882 | -4.7764 | [60] |
|  | Iso-γ-fagarine | *D. dasycarpus* Turcz. | -5.6523 | -5.9337 | [61] |
|  | Isomaculosidine | *D. dasycarpus* Turcz. | -6.1623 | -5.4303 | [61] |
|  | Platydesmine | *D. dasycarpus* Turcz. | -5.8013 | -6.1985 | [59] |
|  | Myrtopsine | *D. dasycarpus* Turcz. | -6.2648 | -5.6199 | [62] |
|  | Deacetyldubinine | *D. dasycarpus* Turcz. | -6.0823 | -5.5917 | [59] |
|  | 3-[1β-Hydroxy-2-(β-D-pyranosyloxy)-ethyl]-4-methoxy-2  (1H)-quinolinone | *D. dasycarpus* Turcz. | -6.2735 | -5.9175 | [61] |
|  | 2(1H)-Quinolinone | *D. dasycarpus* Turcz. | -4.6662 | -3.8591 | [61] |
|  | Preskimmianine | *D. dasycarpus* Turcz. | -6.7404 | -6.3575 | [61] |
|  | Dasycarine | *D. dasycarpus* Turcz. | -7.4321 | -5.9967 | [59], [63] |
|  | 5,9-Dimethoxy-2,2-dimethyl-2H-Pyrano[2,3-b]quinolone | *D. dasycarpus* Turcz. |  |  | [64] |
|  | 3-Chloro-8,9-dimethoxygeibalansine | *D. dasycarpus* Turcz. | -6.5106 | -6.1576 | [64] |
|  | (3R)-3,4-Dihydro-5,8,9-trimethoxy-2,2-dimethyl-2H-Pyrano[2,3-  b]quinolin-3-ol | *D. dasycarpus* Turcz. | -5.8781 | -6.2187 | [64] |
|  | (-)-1’,2-Anhydro-7,8-dimethoxyplatydesmine | *D. dasycarpus* Turcz. | -6.5010 | -6.1336 | [64] |
|  | (S)-7,8-Dimethoxymyrtopsine | *D. dasycarpus* Turcz. | -6.5492 | -6.5123 | [59] |
|  | 7,8-Dimethoxyplatydesmine | *D. dasycarpus* Turcz. | -6.4490 | -6.3493 | [59] |
|  | 1H-indole-3-carboxaldehyde | *D. dasycarpus* Turcz. | -4.8557 | -4.1976 | [65] |
|  | Methyl 4-(2-formyl-5-methoxymethyl1-H-pyrrol-1-yl) butanoate | *D. dasycarpus* Turcz. | -6.6184 | -5.3686 | [65] |
|  | 8-methoxyflindersine | *D. dasycarpus* Turcz. | -5.7587 | -5.3038 | [65] |
|  | 8-methoxy-N-methylflindersine | *D. dasycarpus* Turcz. | -6.4446 | -5.6709 | [65] |
|  | 7-hydroxy-8-methoxydictamnine | *D. dasycarpus* Turcz. | -5.5347 | -5.3988 | [65] |
|  | 1'-oxo-isoplatydesmine | *D. dasycarpus* Turcz. | 6.0577 | -5.1919 | [65] |
|  | Demethoxyacrophylline | *D. dasycarpus* Turcz. | -6.1538 | -5.8557 | [65] |
|  | (+)-Dasycarine A | *D. dasycarpus* Turcz. | -5.9672 | -7.1874 | [66] |
|  | dasycarine B | *D. dasycarpus* Turcz. | -6.6368 | -6.6854 | [66] |
|  | (-)-Dasycarine A | *D. dasycarpus* Turcz. | -6.7092 | -6.7338 | [66] |
|  | dasycarine C | *D. dasycarpus* Turcz. | -6.4275 | -5.8488 | [66] |
|  | dasycarine D | *D. dasycarpus* Turcz. | -6.9853 | -6.3836 | [66] |
|  | dasycarine E | *D. dasycarpus* Turcz. | -7.0469 | -6.1328 | [66] |
|  | Ribalibin | *D. dasycarpus* Turcz. | -5.9594 | -5.0211 | [66] |
|  | Isodictamnine | *D. dasycarpus* Turcz. | -5.1108 | -4.6775 | [66] |
|  | 2,6-dihydro-2,2,7-trimethyl-5H-pyrano[3,2-c]quinolin-5 one | *D. dasycarpus* Turcz. | -6.0282 | -4.7948 | [66] |
|  | 3-quinolinecarboxylic acid | *D. dasycarpus* Turcz. | -4.9373 | -4.5363 | [66] |
|  | 4-methoxy-1H-quinolin-2-one | *D. dasycarpus* Turcz. | -5.0099 | -4.2432 | [66] |
|  | swietenidin B | *D. dasycarpus* Turcz. | -5.3378 | -5.0234 | [66] |
|  | (+)-7,8-dimethoxymyrtopsine | *D. dasycarpus* Turcz. | -6.5008 | -7.1229 | [66] |
|  | 2-hydroxy-4-methoxy-3-(3'-methyl-2'-butenyl)-quinolin | *D. dasycarpus* Turcz. | -5.7027 | -5.4995 | [66] |
|  | Pterygospermin | *Moringa oleifera* Lam. | -7.1028 | -6.9777 | [67] |
|  | O-ethyl-4-(alpha-l-rhamnosyloxy)benzyl carbamate | *M. oleifera* Lam. | -6.7846 | -6.1524 | [67] |
|  | Aurantiamide acetate | *M. oleifera* Lam. | -7.6923 | -7.8413 | [68] |
|  | Marumoside B | *M. oleifera* Lam. | -8.1048 | -7.2790 | [69] |
|  | Marumoside A | *M. oleifera* Lam. | -6.6593 | -5.9864 | [69] |
|  | 1, 3-Dibenzyl urea | *M. oleifera* Lam. | 5.4458 | -5.5612 | [68] |
|  | N,alpha-L-rhamnopyranosyl vincosamide | *M. oleifera* Lam. | -8.8239 | -8.6702 | [70] |
|  | 1-Isopropyl-5-methyl-2-pyrazoline | *M. oleifera* Lam. | -4.4604 | -3.9830 | [71] |
|  | 5,5-Dimethoxypiperidin-2-one | *M. oleifera* Lam. | -4.5114 | -4.2071 | [71] |
|  | 3-Methylquinoline | *M. oleifera* Lam. | -4.9661 | -4.2747 | [71] |
|  | Pyrrolidine, 1-(1,6-dioxooctadecyl)- | *M. oleifera* Lam. | -7.9961 | -6.9241 | [71] |
|  | 2,3-dihydro-4-  methoxy-furo[2,3-  b]quinoline-2,3-  diol | *D. dasycarpus* Turcz. | -5.7921 | -5.3890 | [72] |
|  | 3-(1,2-  dihydroxyethyl)-4-  methoxy-2(1H)-  quinolinone | *D. dasycarpus* Turcz. | -5.6872 | -5.2767 | [72] |
|  | 3,9-dihydro-3-  hydroxy-9-methylfuro[2,3-  b]quinolin-4(2H)-  one | *D. dasycarpus* Turcz. | -5.5919 | -4.8584 | [72] |
|  | 4-methoxyfuro[2,3-  b]quinoline-7,8-  diol | *D. dasycarpus* Turcz. | -5.5885 | -5.2785 | [72] |
|  | furo[2,3-  b]quinolin-4-ol | *D. dasycarpus* Turcz. | -5.1788 | -4.7158 | [72] |
|  | dictangustine A | *D. dasycarpus* Turcz. | -5.4619 | -5.0725 | [72] |
|  | Ribalinine | *D. dasycarpus* Turcz. | -6.2820 | -5.2836 | [72] |
|  | 3-(2-hydroxyethyl)-4-  methoxy-2(1H)-  quinolinone | *D. dasycarpus* Turcz. | -5.5931 | -5.1844 | [72]v |
|  | 7-hydro-8-methoxymyrtopsine | *D. dasycarpus* Turcz. | -6.8453 | -6.1384 | [72] |
|  | Dubinidine | *D. dasycarpus* Turcz. | -6.3708 | -5.8471 | [72] |
|  | Kokusaginin | *D. dasycarpus* Turcz. | -6.1071 | -5.6121 | [72] |
|  | Isopteleine | *D. dasycarpus* Turcz. | -5.5638 | -5.0810 | [72] |
|  | Araliopsinine | *D. dasycarpus* Turcz. | -6.1243 | -5.6353 | [72] |
|  | N-methyl-swietenidine | *D. dasycarpus* Turcz. | -5.6108 | -4.9443 | [72]v |
|  | 4-methoxy-2(1H)-  Quinolinone | *D. dasycarpus* Turcz. | -5.0649 | -4.3105 | [72] |
|  | 5-  Hydroxydictamnin | *D. dasycarpus* Turcz. | -5.3395 | -4.8129 | [72] |
|  | 3-acetyl-4-  methoxy-2(1H)-  quinolinone | *D. dasycarpus* Turcz. | -5.4909 | -4.7631 | [72] |
