# Supplementary figures and images for: Identifying plant-derived antiviral alkaloids as dual inhibitors of SARS-CoV-2 main protease and spike glycoprotein through computational screening
Source: Front Pharmacol. 2024 Jul 17;15:1369659. doi: 10.3389/fphar.2024.1369659 (PMC11288853; doi:10.3389/fphar.2024.1369659)

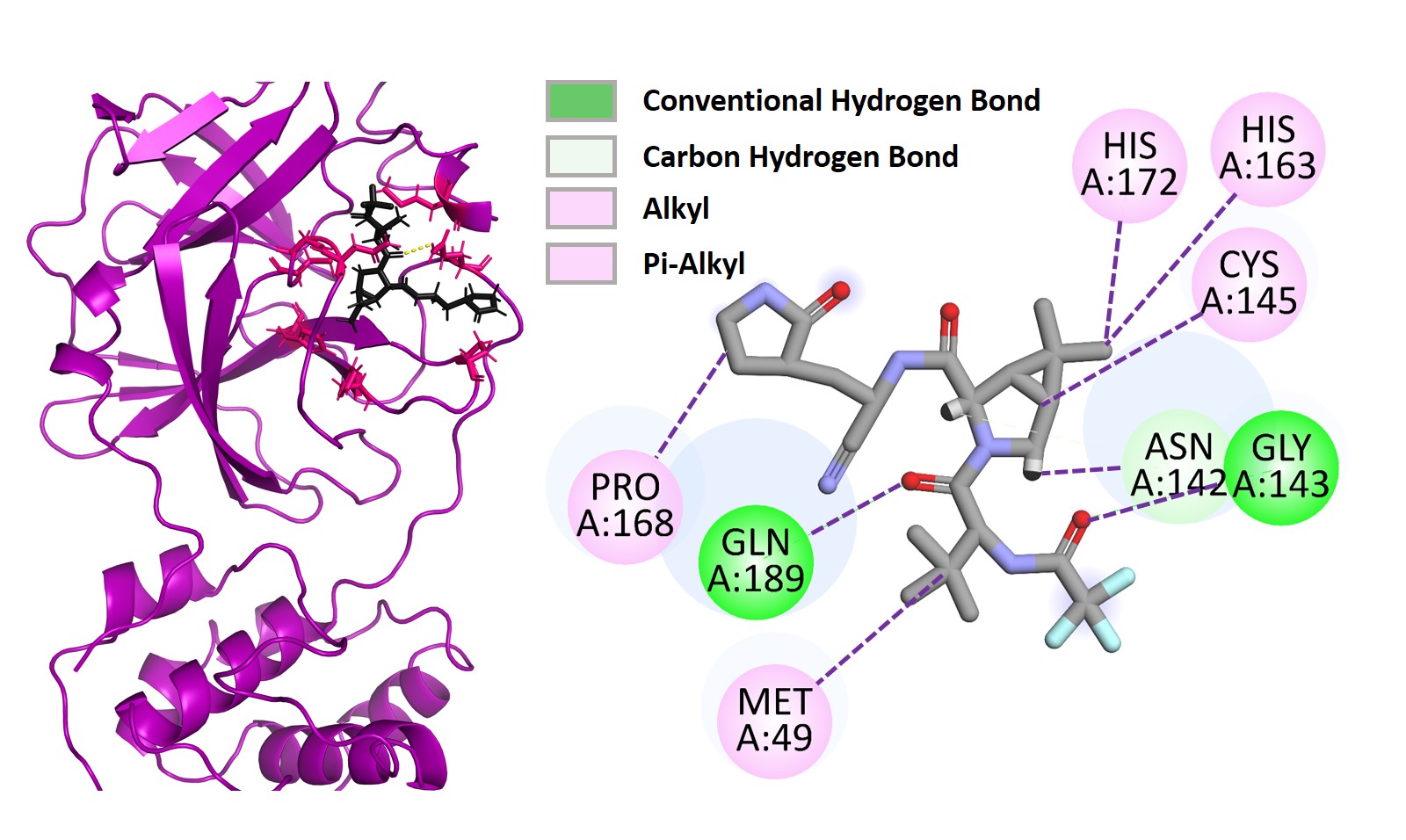

Supplement: Supplementary file 2 [file Image1.JPEG]

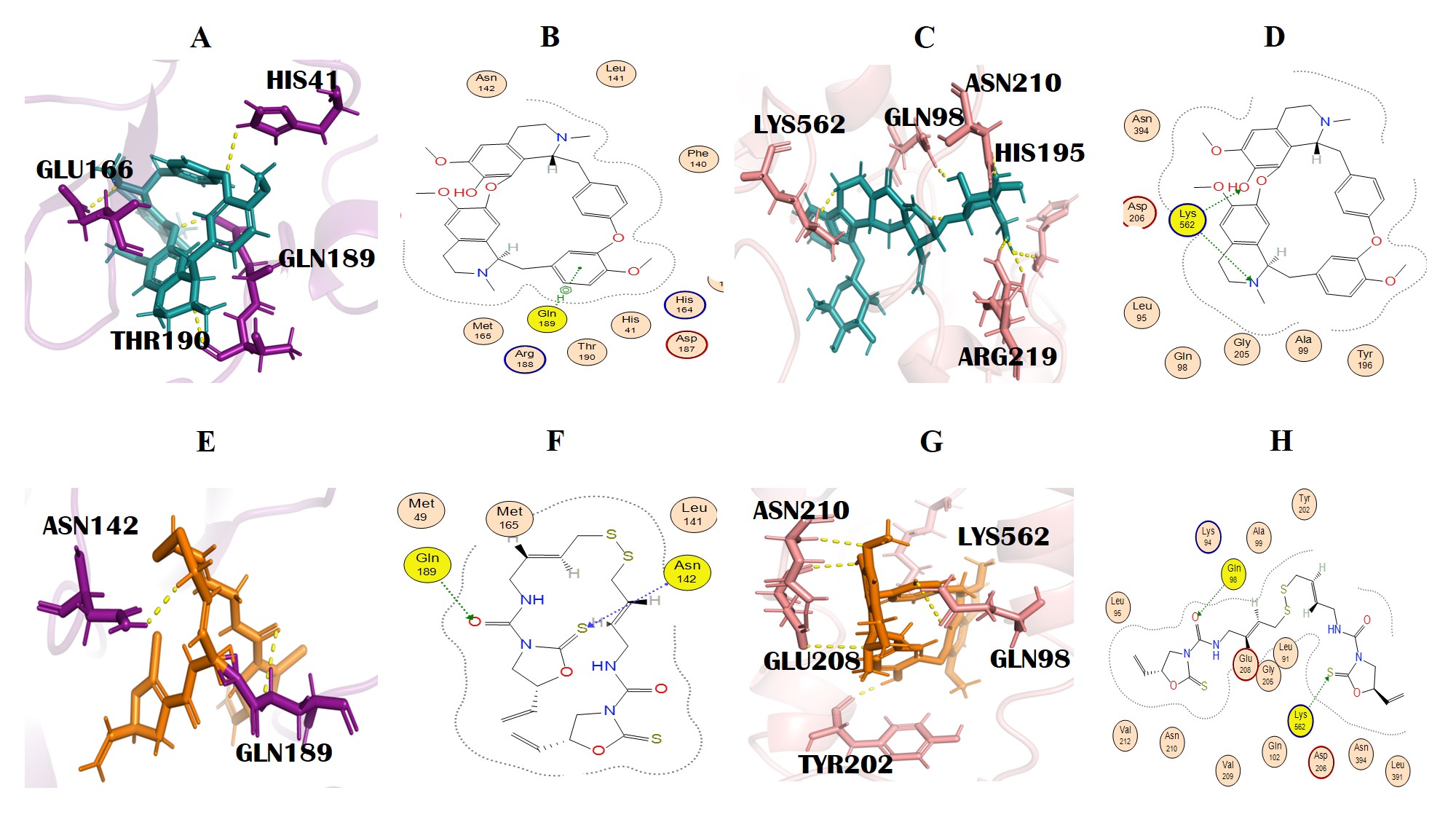

Supplement: Supplementary file 3 [file Image2.JPEG]
