# Supplementary material for: Identifying plant-derived antiviral alkaloids as dual inhibitors of SARS-CoV-2 main protease and spike glycoprotein through computational screening
Source: Front Pharmacol. 2024 Jul 17;15:1369659. doi: 10.3389/fphar.2024.1369659 (PMC11288853; doi:10.3389/fphar.2024.1369659)
Supplement: Supplementary file 4 [file Table2.docx]

**Table S2.** Interactions of dual active alkaloids with Main protease.

| Alkaloid Name | Main Protease interacting residues | Bond type | Distance | Energy | Residues Involved In hydrophobic interaction |
| --- | --- | --- | --- | --- | --- |
| Adouetine Y | Glu166 | H-acceptor | 2.86 | -2.8 | Thr25, Thr26, His41, Met49, Leu141, Asn142, Gly143 Cys145, Met165, Pro168, Arg188, Thr190, and Gln192. |
|  | Gln189 | H-acceptor | 2.39 | -1.7 |  |
| Ergosine | Met49 | H-donor | 3.67 | -0.3 | Thr25, Thr26, Leu27, His41, Ser46, Met49, Cys145, Glu166, Asp187, Arg188, and Gln189. |
|  | Met49 | H-donor | 3.53 | -0.8 |  |
|  | Asn142 | H-acceptor | 2.81 | -0.9 |  |
|  | Gln189 | pi-H | 4.05 | -0.8 |  |
| Evodiamide C | Thr25 | pi-H | 3.73 | -1.0 | Thr24, Thr25, Thr26, His41, Thr45, Met49, Phe140, Asn142, Ser144, Glu166, Asp187, Arg188, Gln189, Thr190, Gln192, His163, Met165, and Pro168. |
|  | Ser46 | H-acceptor | 3.29 | -1.9 |  |
|  | Cys145 | H-donor | 3.25 | -1.5 |  |
|  | Cys145  His164 | H-donor  H-bond | 3.48  2.2 | -1.4  --- |  |
|  | Gln189 | pi-H | 4.11 | -2.2 |  |
|  | Gln189 | pi-H | 4.50 | -0.9 |  |
| Reserpine | Asn142  Thr190  Gln192 | H-acceptor  H-bond  H-bond | 2.83  2.50  2.00 | -3.1  ---  --- | His41, Met49, Asn142, His164, Met165, Glu166, Pro168, Asp187, Gln189, Thr190, and Gln192. |
| Pelosine | Glu166  Glu166  Arg188 | H-bond  H-bond  H-bond | 3.40  2.60  2.70 | ---  ---  --- | Thr25, Thr26, Leu27, His41, Val42, Met49, Leu141, Gly143, Cys145, His164, Glu166, Arg188, and Gln189. |
| Hayatinine | Thr190  Thr190  Gln192 | H-bond  H-bond  H-bond | 2.20  2.40  2.60 | ---  ---  --- | Thr25, Thr26, His41, Thr45, Ser45, Met49, Pro52, Phe140, Leu141, Gly143, Ser144, Cys145, His163, His164, Met165, Glu166, Leu167, Pro168, Asp187, Arg188, Gln189, Thr190, and Gln192. |
| Homoarmoline | His41  Glu166  Gln189  Thr190 | H-bond  H-bond  pi-H  H-bond | 2.80  2.70  4.28  2.40 | ---  ---  -0.6  --- | Thr25, Met49, Leu141, Asn142, Cys145, His164, Met165, Glu166, Pro168, Asp187, Arg188, Gln189, Thr190, and Gln192. |
| Isatithioetherine C | Asn142 | H-acceptor | 3.81 | -0.9 | Thr25, Thr26, His41, Met49, Leu141, Asn142, His164, Met165, Glu166, Asp187, Arg188, and Gln192. |
|  | Gln189 | H-acceptor | 3.08 | -0.7 |  |
| N,alpha-L-rhamnopyranosyl vincosamide | Thr26  Met49 | H-donor  H-donor | 3.11  3.63 | -0.8  -0.8 | Thr25, Leu27, His41, Met69, Asn142, Met165, Glu166, Pro168, Gln189, and Thr190. |
|  | Gly143 | H-acceptor | 3.05 | -1.7 |  |
| Toddalidimerine | Glu166 | pi-H | 4.67 | -0.6 | Thr25, His41, Ser46, Met49, Leu50, Leu141, Asn142, Cys145, Met165, Glu166, Leu167, Pro168, Arg188, and Thr190. |
| Toddayanis | His163 | H-acceptor | 2.91 | -0.9 | Thr25, His41, Met49, Phe140, Leu141, Asn142, Gly143, Ser144, Cys145, His164, Met165, Glu166, Pro168, His172, Asp187, Arg188, Thr190, and Gln192. |
| Zanthocadinanine | Gln189 | pi-H | 4.20 | -0.8 | Thr25, Thr26, His41, Ser46, Met49, Leu141, Asn142, Gly143, Met165, Glu166, Asp187, Arg188, and Gln189. |
