# Supplementary material for: Identifying plant-derived antiviral alkaloids as dual inhibitors of SARS-CoV-2 main protease and spike glycoprotein through computational screening
Source: Front Pharmacol. 2024 Jul 17;15:1369659. doi: 10.3389/fphar.2024.1369659 (PMC11288853; doi:10.3389/fphar.2024.1369659)
Supplement: Supplementary file 5 [file Table3.docx]

**Table S3**. Interactions of dual active alkaloids with Spike Glycoprotein

| **Alkaloid Name** | **Interacting residues** | **Bond type** | **Distance** | **Energy**  **kcal/mol** | **Residues involved in hydrophobic interaction** |
| --- | --- | --- | --- | --- | --- |
| Adouetine Y | Gln98 | H-donor | 3.10 | -2.66 | Lys94, Leu95, Tyr196, Trp203, Gly205, AAsp206, Glu208, Asn210, Glu398, Lys562, and Pro565. |
|  | Gln98 | H-acceptor | 2.66 | -1.2 |  |
| Ergosine | Leu95 | H-bond | 2.40 | --- | Leu95, Tyr196, Try202, Trp203, Asp206, Glu208, Asn210, and Glu398 |
|  | Gln98 | H-acceptor | 3.24 | -0.8 |  |
|  | Gly102 | H-acceptor | 3.04 | -0.8 |  |
|  | Gly205 | H-donor | 3.02 | -3.3 |  |
|  | Asn210 | H-bond | 2.20 | --- |  |
|  | Lys562 | H-acceptor | 3.26 | -1.1 |  |
| Evodiamide C | Gln98 | H-bond | 1.80 | --- | Leu91, Lys94, Leu95, Ala99, Tyr202, Trp203, Gly205, Asp206, Glu208, Val209, Asn210, Gly211 Val212, Leu391, Glu398, and Lys562. |
|  | Gln102 | H-bond | 2.50 | --- |  |
|  | Thr196 | H-bond | 2.40 | --- |  |
|  | Gly205 | H-bond | 1.90 | --- |  |
|  | Lys562 | H-bond | 2.90 | --- |  |
|  | Lys562 | Pi-cation | 3.94 | -1.3 |  |
| Reserpine | Gln208 | H-bond | 2.80 | --- | Leu91, Lys94, Leu95, Ala99, Trp203, Gly205, Asp206 Glu208, Val209, Leu391, Ala396, Glu398, Lys562, and Trp566. |
|  | Asn210 | H-bond | 2.95 | -3.1 |  |
|  | Lys562 | H-bond | 3.00 | --- |  |
| Pelosine | Tyr202 | H-donor | 2.95 | -1.0 | Leu95, Gln98, Ala99, Gln102, Tyr196, Tyr202, Trp203, Gly205, Asp206, Val209, Asn210, Arg219, Leu391, Asn394, Glu398, and Lys562. |
| Hayatinine | Gln98 | H-donor | 2.10 | --- | Leu95, Gln98, Ala99, Gln102, Tyr196, Tyr202, Trp203, Gly205, Asp206, Val209, Arg219, Leu391, Asn394, Glu398, and Lys563. |
|  | Lys562 | Pi-cation | 3.24 | -1.3 |  |
| Homoarmoline | Gln98 | H-bond | 2.00 | --- | Leu95, Ala99, Gln102, Tyr196, Tyr202, Trp203, Gly205, Asp206, Leu391, and Glu398. |
|  | His195 | H-bond | 2.40 | --- |  |
|  | Asn210 | H-bond | 2.50 | --- |  |
|  | Arg219 | H-bond | 2.30 | --- |  |
|  | Arg219 | H-bond | 2.70 | --- |  |
|  | Lys562 | H-acceptor | 3.05 | -1.4 |  |
| Isatithioetherine C | Gln98 | H-acceptor | 3.06 | -1.5 | Leu91, Leu95, Ala99, Gln102, Tyr202, Trp203, Gly205, Asp206, Glu208, Asn210, Leu391, Asn394, and Glu398. |
|  | Tyr202 | H-bond | 1.90 | --- |  |
|  | Glu208 | H-bond | 2.40 | --- |  |
|  | Glu208 | H-bond | 3.40 | --- |  |
|  | Asn210 | H-bond | 3.60 | --- |  |
|  | Lys562 | H-acceptor | 4.03 | -6.9 |  |
| N,alpha-L-rhamnopyranosyl vincosamide | Gln98 | H-donor | 2.66 | -1.3 | Leu91, Lys94, Leu95, Ala99, Tyr196, Tyr202, Trp203, Gly205, Val209, Leu392, Asn394, Glu398, Ser563, Glu564, Pro565, and Trp566. |
|  | Gln102 | H-acceptor | 3.19 | -1.4 |  |
|  | Asn210 | H-bond | 2.20 | --- |  |
|  | Lys562 | H-acceptor | 3.12 | -3.5 |  |
| Toddalidimerine | Gln98 | H-bond | 2.60 | --- | Leu95, Ala99, Gln102, Tyr196, Tyr202, Trp203, Gly205, Asp206, Glu208, Asn210, Leu391, Leu392, Ala396, Glu398, Lys562, Glu564, Pro565, and Trp566. |
|  | Val209 | Pi-H | 4.49 | -0.6 |  |
|  | Lys562 | Pi-cation | 3.34 | -0.9 |  |
| Toddayanis | Gln98 | H-acceptor | 2.62 | -0.9 | Leu95, Ala99, Gln102, Tyr196, Tyr202, Trp203, Gly205, Asp206, Glu208, Val209, Asn210, Leu391, Leu392, Asn394, Glu398, and Lys562. |
|  | Lys562 | Pi-cation | 3.57 | -1.7 |  |
| Zanthocadinanine | Gln102 | H-bond | 1.70 | --- | Leu91, Lys94, Leu95, Gln98, Ala99, Gln102, His195,Tyr196, Val209, Asn210, Tyr202, Trp203, Gly205, Asp206, Glu208, Val212, Arg219,Leu392, Asn394, Glu398, Lys562, Lys562, Ser563, Glu564, Pro565, and Trp566. |
|  | Tyr196 | H-bond | 2.60 | --- |  |
|  | Asn210 | Pi-H | 4.34 | -0.6 |  |
