# Supplementary material for: Identifying plant-derived antiviral alkaloids as dual inhibitors of SARS-CoV-2 main protease and spike glycoprotein through computational screening
Source: Front Pharmacol. 2024 Jul 17;15:1369659. doi: 10.3389/fphar.2024.1369659 (PMC11288853; doi:10.3389/fphar.2024.1369659)
Supplement: Supplementary file 6 [file Table4.docx]

| **Sr. No.** | **Name** | **MW**  **(g/mol)** | **Log P** | **nRB** | **nHBA** | **nHBD** | | **TPS** | **MR** |
| --- | --- | --- | --- | --- | --- | --- | --- | --- | --- |
| 1 | Adouetine Y | 568.71 | 3.61 | 9 | 5 | 3 | 99.77 | | 172.11 |
| 2 | Ergosine | 547.65 | 1.93 | 5 | 6 | 3 | 118.21 | | 160.28 |
| 3 | Evodiamide C | 656.69 | 3.36 | 8 | 6 | 2 | 160.19 | | 191.27 |
| 4 | Hayatinine | 608.72 | 5.12 | 3 | 8 | 1 | 72.86 | | 181.60 |
| 5 | Homoarmoline | 608.72 | 5.18 | 3 | 8 | 1 | 72.86 | | 181.60 |
| 6 | Isatithioetherine C | 514.70 | 2.89 | 15 | 4 | 2 | 197.92 | | 145.47 |
| 7 | N,alpha-L-rhamnopyranosyl vincosamide | 660.67 | -1.13 | 6 | 13 | 7 | 213.00 | | 163.23 |
| 8 | Pelosine | 594.70 | 4.84 | 2 | 8 | 2 | 83.86 | | 177.14 |
| 9 | Reserpine | 608.68 | 3.52 | 10 | 10 | 1 | 117.78 | | 165.52 |
| 10 | Toddalidimerine | 738.78 | 6.41 | 8 | 9 | 1 | 106.18 | | 214.84 |
| 11 | Toddayanis | 569.73 | 6.21 | 5 | 5 | 1 | 60.39 | | 172.22 |
| 12 | Zanthocadinanine | 5.83.76 | 6.65 | 6 | 5 | 0 | 49.39 | | 179.95 |

**Table S4.** Physicochemical properties of dual-active alkaloids.

MW (Molecular weight), nRB (Number of rotatable bond), nHBA (No of hydrogen bond acceptor), nHBD (No of hydrogen bond donor), TPS (Topological surface area) and MR (Molar refractivity)
